# Canalization of the Polygenic Risk for Common Diseases and Traits in the UK Biobank Cohort

**DOI:** 10.1093/molbev/msac053

**Published:** 2022-03-11

**Authors:** Sini Nagpal, Raghav Tandon, Greg Gibson

**Affiliations:** 1 School of Biological Sciences, and Center for Integrative Genomics, Georgia Institute of Technology, Atlanta, GA, USA; 2 Wallace H. Coulter Department of Biomedical Engineering, and Center for Machine Learning, Georgia Institute of Technology, Atlanta, GA, USA

**Keywords:** polygenic score, PGS×E interaction, epistasis, metabolic disease, inflammatory bowel disease, educational attainment

## Abstract

Since organisms develop and thrive in the face of constant perturbations due to environmental and genetic variation, species may evolve resilient genetic architectures. We sought evidence for this process, known as canalization, through a comparison of the prevalence of phenotypes as a function of the polygenic score (PGS) across environments in the UK Biobank cohort study. Contrasting seven diseases and three categorical phenotypes with respect to 151 exposures in 408,925 people, the deviation between the prevalence–risk curves was observed to increase monotonically with the PGS percentile in one-fifth of the comparisons, suggesting extensive PGS-by-Environment (PGS×E) interaction. After adjustment for the dependency of allelic effect sizes on increased prevalence in the perturbing environment, cases where polygenic influences are greater or lesser than expected are seen to be particularly pervasive for educational attainment, obesity, and metabolic condition type-2 diabetes. Inflammatory bowel disease analysis shows fewer interactions but confirms that smoking and some aspects of diet influence risk. Notably, body mass index has more evidence for decanalization (increased genetic influence at the extremes of polygenic risk), whereas the waist-to-hip ratio shows canalization, reflecting different evolutionary pressures on the architectures of these weight-related traits. An additional 10 % of comparisons showed evidence for an additive shift of prevalence independent of PGS between exposures. These results provide the first widespread evidence for canalization protecting against disease in humans and have implications for personalized medicine as well as understanding the evolution of complex traits. The findings can be explored through an R shiny app at https://canalization-gibsonlab.shinyapps.io/rshiny/.

## Introduction

Organisms evolve and develop in fluctuating environments. From the species’ perspective, it makes sense that genetic architectures should ensure that as many individuals resist these fluctuations and are as close to maximal fitness as possible. Yet individuals must cope with the vicissitudes of life—the slings and arrows of outrageous fortune—while facing the challenges of staying fit. Thus, the concept of the optimum trait is a useful abstraction that belies the reality that genetics must also support both flexibility and robustness. The evolution of robustness is known as canalization (Gibson and Wagner 2000; Flatt 2005), but this phenomenon remains relatively understudied, and there is little global evidence pertaining to the prevalence and its importance.

The individuals in a population who are at the highest risk of abnormality are those who have the greatest genetic liability to deviate from the mean. They are the ones least likely to be able to absorb the effects of pathogenic de novo mutations or inherited rare variants; they are the ones most at risk in unhealthy environments. In light of genome-wide association studies (GWASs), the identification of such individuals has become straightforward for dozens of human diseases since polygenic scores (PGSs, also known as polygenic risk scores, PRSs, in the context of disease) allow the ranking of individuals with respect to the likelihood of having the condition (Dudbridge 2013; Duncan et al. 2019). Each of us is in the top few percentiles of the PRS for some disease, typically being 3- to 5-fold more likely to have it than the average person (Khera et al. 2018). The question arises as to how great the relative risk is for those in the top percentiles who also have unhealthy environmental exposures. Technically speaking, are there PGS-by-Environment (PGS×E) interactions that moderate the genetic liability at the extremes of the distribution?

Two possibilities with respect to the presence of canalization can be envisaged. The first is decanalization (Gibson 2009), namely exacerbated prevalence in individuals with high polygenic risk. A mechanism for this would be an inability to compensate for the combined effects of genetic and environmental perturbation, essentially a loss of titration capacity of the genetic architecture (Rice 1998). The converse possibility is that genetic systems are, in fact, sufficiently well canalized that they are able to buffer environmental effects (Wagner et al. 1997), ensuring that fewer individuals have the pathogenic state than expected given the environmental deviation. Comparative analysis of prevalence–risk curves across environments in large cohort studies provides an opportunity to evaluate the presence of either or both scenarios in human populations.

Either canalization or decanalization would be revealed as a departure from the null expectation that the two curves are similar in two subsets of a cohort defined by different exposures. However, there are actually two models for the null, which complicates the comparison. A prevalence–risk curve plot observed prevalence against percentile of risk. It typically has an inverted S distribution seen in many figures in this study, being concave in the bottom half and convex in the top half. Most individuals have approximately the same prevalence, but those with low risk are well protected, and those at high risk appear to have much-elevated prevalence. The simplest conception of the effect of an unhealthy environment would be that it increases the prevalence by a similar amount for all percentiles: this would imply an additive effect of environment plus PGS, and the two curves are just shifted along the *y*-axis with respect to one another. However, that null is not compatible with the conception of the PGS as a sum of the logarithms of the odds ratios of individual SNPs, in which case the score multiplies the average risk (i.e., multiplicative allelic effects are assumed to be additive on the log scale, but their effect is relative to the population mean). If the environment increases average risk, then two prevalence–risk curves would be expected to deviate from each other to an ever-increasing extent as the risk increases. Consequently, allelic substitution effects appear to get larger in the poor environment as the PGS increases, implying that the genetic variance is greater at the extreme—which paradoxically is the expectation under decanalization. Furthermore, odds ratios are a function of the overall prevalence, which is elevated in a harsher environment, further complicating the direct comparison of the deviation between the curves in two environments.

Here we report on the prevalence of PGS×E interactions for ten complex traits in the UK Biobank (UKB) cohort study of over 400,000 White British persons with data on 151 exposures (Sudlow et al. 2015). He et al. (2019), expanding on Rask-Andersen et al. (2017), showed that exposure effects are often at least as predictive of disease as a polygenic risk in this cohort but did not examine their interaction with genetics. Conversely, a sophisticated likelihood modeling approach recently found evidence that PGS×E interactions explain several percent of BMI-associated traits (Sulc et al. 2020) without actually identifying the specific environmental factors. We develop a more empirical modeling approach that allows decomposition of the additive and log-additive contributions to the deviance between prevalence–risk curves in different environments. Increasing deviation with polygenic risk is pervasive and has a marked impact on individual predictions, with implications for predictive health. Across the data set, although, we find that in general that the degree of robustness of polygenic risk to environmental differences is very much trait-specific. These data imply that the human genome sometimes exhibits canalization that buffers the impact of unhealthy environments, restricting the impact of modern lifestyles that promote decanalization but also identifies situations where lifestyle exacerbates genetic risk synergistically.

## Results

### Prevalence–Risk Relationships in the UKB

**Fig. 1.**
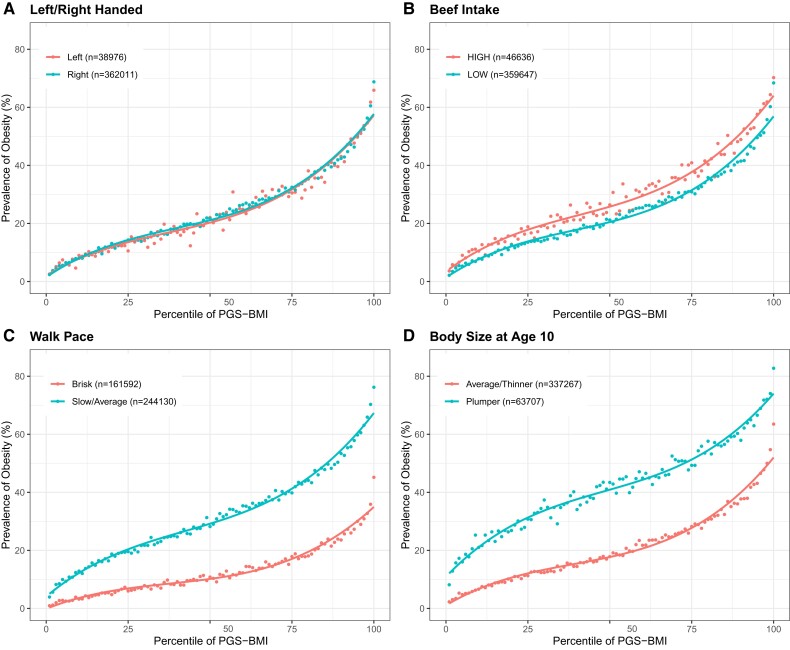
Four classes of prevalence versus polygenic risk relationships. Each panel shows two curves where the dots represent the observed prevalence of obesity for the percentile of PGS score for BMI in the high-risk or low-risk environment. (*A*) No effect (left vs. right-handed). (*B*) Constant shift (high vs. low beef intake). (*C*) Decanalization as defined in text (walk pace, slow/average vs. brisk). (*D*) Nonmonotonic change (body size at age 10, plumper vs. thinner).

We begin by asking whether there is a difference in the shape of the prevalence–risk curve for each of the ten traits considered in two subsets of the UKB cohort differentiated with respect to exposure. Figure 1 illustrates four classes of results for the prevalence of obesity (BMI >30) as a function of the PGS_BMI_ built from 11,445 LD-pruned SNPs significant for BMI at *P* < 0.001. No difference is seen when the cohort is divided into two halves with respect to self-reported left-or-right handedness in fig. 1(*A*). The two curves overlay almost perfectly, showing that ∼ 20% of people with intermediate polygenic risk for BMI are obese, whereas the prevalence of the condition is at least 3-fold lower (<5%) or higher (>60%) in the bottom and top percentiles, respectively. Next, individuals who report high beef intake are uniformly 2–3% more likely to be obese in fig. 1(*B*), largely irrespective of their polygenic risk since the high beef intake curve is essentially displaced upward relative to the low one. This is an example of an additive PGS + E effect. In contrast, in fig. 1(*C*) shows an instance where the effect of walking pace has an increasingly large impact on the prevalence of obesity across the PGS spectrum. This measure of physical fitness has little impact on obesity for those with very low polygenic risk who are unlikely to be obese, but as the PGS percentile increases, so does the deviation between the curves, with the consequence that there is a 20% difference in prevalence for intermediate PGS and as much as 30% increase for slow walkers. This is an example of PGS×E interaction, on the face of it explained by log-additive effects of genotype modifying different baseline risks in the low- and high-activity subsets. The fourth type of result shown in fig. 1(*D*) for body size at age 10 (self-reported as plumper or thinner) involves a nonmonotonic change in the slopes of the curves, which are most divergent for intermediate risk. In this case, plumper kids appear to have an elevated risk of developing adult obesity in the midrange of genetic risk, as the two curves converge at the two extremes. Examples of this mode of canalization, and other noncanonical deviations, are rare in the total data set.

To qualify provisionally as an instance of PGS×E, we required that two conditions be met: the first is that the first derivative of the difference in the prevalence of the two curves along with the PGS percentile be consistently either greater or lesser than zero, and the second is that the linear deviation in slope be greater than that seen in 100 permutations of the labels for the two exposures (see Materials and Methods). The first requirement ensures that the polygenic effect is always increasing in one of the two environments as the PGS percentile increases, and the second ensures that the effect is greater than expected by chance. The ten traits are binary categorical evaluations of obesity as a function of body mass index (BMI) and waist-to-hip ratio (WHR), coronary artery disease (CAD), type 2 diabetes (T2D), depression (DEP), college attainment (CA), and inflammatory bowel disease (IBD), as well as three continuous trait evaluations of BMI, WHR, and educational attainment (EA) in years. The 151 exposures were considered in seven categories, namely socioeconomic, early life, psychosocial, familial factors, diet, lifestyle, and general health. Within these categories, there is often good agreement for similar measurements: walking pace, duration of exercise, and the number of stairs climbed per day typically give the same conclusion, implying that they capture some underlying lifestyle risk factor. On the other hand, there are also some notable exceptions, such as differences in outcome with respect to beer, wine, and spirit consumption as measures of alcohol intake. Supplementary table S1, Supplementary Material online lists the complete matrix of results, and we have generated a Shiny app for exploration of the results, including an additional explanation of the methods: https://canalization-gibsonlab.shinyapps.io/rshiny/

**Fig. 2.**
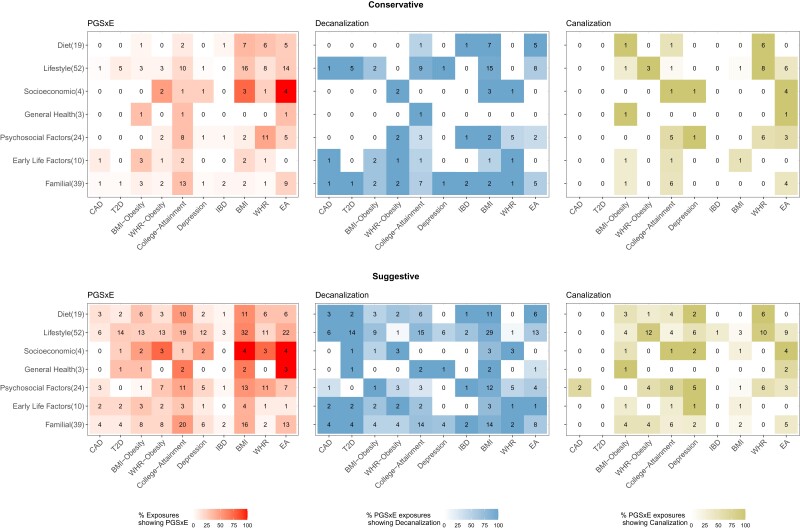
Percentages of apparent PGS×Environment interaction per trait by type of exposure. Digits in each box indicate the number of exposures in each class significant at conservative or suggestive cutoffs defined in the Materials and Methods. See supplementary table S1, Supplementary Material online for the actual exposures. Shading represents the percentage of exposures showing PGS×E or of the PGS×E due to decanalization or canalization, as indicated.

The extent of PGS×E across the study is summarized in fig. 2, which displays the percentage of instances for each trait by category combination after additional filtering to more robustly define cases of canalization and decanalization as described in subsequent sections. Overall, one-fifth of all tests yield suggestive evidence for these PGS×E interactions, with particular enrichment for lifestyle (e.g., exercise, smoking, and drinking) and familial (parental and sibling health) factors. Among the binary conditions, obesity, and T2D have more instances of polygenic risk being modified by the environment than CAD or IBD. The continuous traits have substantial evidence for PGS×E, although this may be due to different definitions. Notably, PGS_WHR_ has more instances of apparent canalization than PGS_BMI_, consistent with evidence that different genetic risk factors influence obesity through WHR and BMI (Nettleton et al. 2015; Loos 2018). An additional 12% of cases show evidence for an additive shift of the curve irrespective of environment, most notably for WHR for which almost half of the exposures have an effect with little evidence for PGS×E.

### Notable Environmental Interactions with Disease and EA

Certain results pertaining to the prevalence of IBD, as well as the subtypes ulcerative colitis (UC) and Crohn’s disease (CD) illustrate environmental exposures that have been implicated in the disease (Carbonnel et al. 2009) but not before shown to interact with genetic risk (supplementary fig. S1, Supplementary Material online). Regarding dietary factors, bread type (but not bread intake) shows a strong effect for both UC and CD, where preference for whole grain over white bread is associated with dampening of the PGS effect. Intriguingly, this preference was found to be the major driver of PC1 of dietary habits in the UKB (Cole et al. 2020). Similarly, reduced salt intake seems to be protective only for individuals at high genetic risk, as is fresh fruit consumption for CD—but not for UC. Past tobacco smoking is confirmed as an important environmental risk factor for all IBD (Jones et al. 2020), whereas intake of alcohol mildly interacts preferentially with CD risk (Jones et al. 2020; Di Narzo et al. 2021). Most of these results were confirmed with two different sets of GWAS effect sizes reported at different stages of IBD GWAS studies and with SNP inclusion thresholds of 5×10^−8^ or 10^−3^. Otherwise, PGS×E are much less frequent than those observed for chronic metabolic diseases, CAD, T2D, and obesity.

CA (Lee et al. 2018) is notable for the very strong interaction of sociodemographic factors with polygenic risk (supplementary fig. S2, Supplementary Material online). The Townsend deprivation index shows a particularly interesting interaction, whereby the frequency of participants who obtain a college degree is ∼35% irrespective of the index but shows a strong apparent buffering effect at both extremes: individuals in the bottom quintile of the EA PGS are actually consistently 3% less likely to attain a college degree if they are in the top third of Townsend deprivation, whereas those in the upper quintile are consistently 3% more likely to attain one. This is a classic example of decanalization, where worsened socioeconomic conditions (high Townsend scores) are the perturbation leading to more variance in EA. A similar result has been described for adoption, where adopted children with low PGS_EA_ have an elevated prevalence of college education (Cheesman et al. 2020). Maternal smoking at birth has a mild tendency to suppress the advantage of polygenic propensity across the range of scores, preference for whole grain bread, and high cheese consumption associated with enhanced genetic effects, but both poultry and beef intake also appear to suppress them. Larger families also show increased prevalence at the upper extreme, although this and the dietary results may be consequential rather than causal, namely elevated PGS_EA_ increase the likelihood of higher education which then increases the resources that facilitate changes in the environmental factors. Indirect genetic effects are well recognized to influence GWAS estimates but are rarely acknowledged for their contribution (Young et al. 2019). College-educated UKB participants are more likely to consume wine or use a computer but less likely to consume beer or cider or to report watching a lot of TV, in each case showing extensive PGS×E interactions. A number of factors interact with PGS_EA_ only over the bottom half of the score, whereas the curves are simply parallel in the top half, including smoking.

**Fig. 3.**
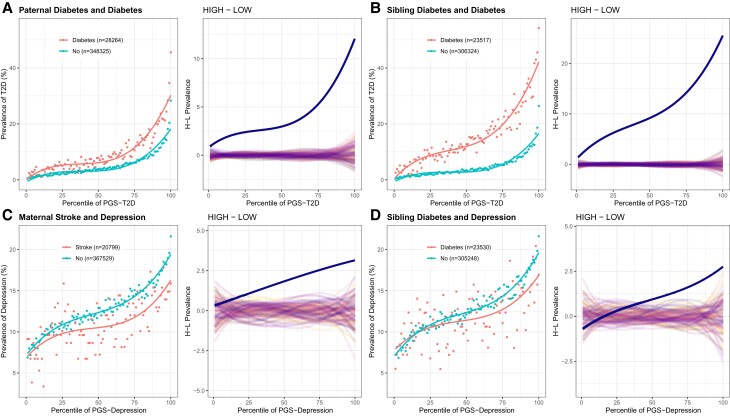
Prevalence–risk curves for familial influences on complex disease risk. (*A* and *B*) Paternal and sibling diabetes shows PGS×E, which suggests decanalization of T2D prevalence but likely reflects an effect of shared genetics not captured by the PGS. (*C* and *D*) Severe maternal or sibling disease influences the prevalence of major DEP in an apparently protective manner. In each panel, the left-hand plot shows the prevalence–risk curve in the exposure group with the indicated numbers of individuals. The right panel shows the difference between the two curves in blue relative to computations based on 100 permutations of exposure labels.

It is also striking that several familial factors influence more than half of the traits, generally exacerbating the polygenic risk. Mother, father, or sibling having any illness, for example, tends to interact with PGS for T2D (with or without obesity), CAD, BMI, and EA, and as shown in fig. 3, the familial incidence of diabetes dramatically influences the relationship. This might be explained by excess sharing of diabetes risk within families (both genetic and environmental) that is not captured by the PRS derived from analysis of unrelated individuals (Muñoz et al. 2016; Kong et al. 2018). Intriguingly, the impact of a severe illness is inverted for DEP as, for example, having a mother who has suffered a stroke or a sibling with diabetes tends to suppress the impact of genetics. Such a result warrants further exploration but might be explained by the act of caregiving providing meaning to life that offsets otherwise present tendencies toward experiencing major DEP.

It is important to note that a simple comparison of prevalence–risk curve differences does not establish causality. For certain traits where the exposure precedes the onset of the condition, such as breastfeeding as a baby decreasing the polygenic contribution to T2D and CAD, causality is likely. For others, such as “time spent outdoors in summer” or “daytime dozing,” it seems more likely that the onset of disease increases the likelihood of a switch in the exposure, contributing to steeper prevalence–risk curves. For some, if not most, traits, the specific exposure—aspect of diet or exercise, for example—should be regarded as a proxy that captures an unmeasured liability, often related to socioeconomic status, which may have been present before the onset of disease.

### Modeling the Deviation between Prevalence–Risk Curves

Under the threshold liability model of disease, an individual’s likelihood of being a case increases as their PGS increases, and the observed prevalence in each percentile of the PGS defines the likelihood of being a case. As the heritability explained by SNPs increases, so does the slope of the prevalence–risk curve. PRSs follow a normal distribution under the genetic liability model, and the characteristic S-shape is expected for a normally distributed score. The precise degree of curvature at either end is a function of the relationship between the PGS and risk and of the variance of the PGS distribution, but there is surprisingly little theory relating the prevalence of disease to PRSs (Slatkin 2008; Wray and Goddard 2010; Agarwala et al. 2013).

On the assumption that the PGS accurately represents the summation of the log of odds ratios (Dudbridge 2013), prevalence should simply be the mean risk multiplied by the PGS for the percentile. However, modeling the deviations between the curves observed between environments allows for quantification of whether there is attenuation of risk, as calibration of the PGS is needed to produce a good fit between observed and predicted data. We model the likelihood that an individual has a condition as a function of three parameters: a modifiable baseline (MBL) probability that is acted upon by the PGS, a calibration factor (CF) that scales the variance of the PGS to the environment, and a setpoint (SP) that adjusts for risk independent of genetics. In addition, a random environmental deviation adjusts for the proportion of variance explained by genotypes:

OverallRisk=SP+MBL×exp(PGS/CF)+Env

Here, the nonmodifiable (SP) and modifiable baselines (MBL) sum to yield the median risk, and the calibration factor (CF), by modifying the PGS, effectively shapes the curvature of the prevalence–risk curve. The environmental term is assumed to be normally distributed with a mean of zero. The PGS has an observed standard deviation (SD), and after scaling to a median of zero, the exponential term is unity at the 50th percentile of risk, and the SP provides a linear adjustment that ensures the overall risk is as observed. More details are provided in Materials and Methods.

If genetic effects are the same across environments with similar prevalence (Duncan et al. 2019), the CF should be the same, whereas a reduction in the CF will generally increase the curvature, enhancing the monotonic increase in the deviation between the curves. Under some circumstances, the deviation may be explained simply by a shift in the SP; for example, adding kindergarten as a year of education may increase EA for everyone regardless of genetics. In such cases, there is no expectation that genetics would modify the baseline risk, and the curves with and without kindergarten in the computation would show a simple shift similar to the one in fig. 1*B*. However, environmental changes are more generally expected to change the baseline that is modified by genetics; for example, opening a new technical college in a district is likely to raise attainment only for a subset of individuals. The increased deviation between the risk–prevalence curves is expected as MBL increases. The question is, although, whether this increase is greater or lesser than expected without modifying the CF.

**Fig. 4.**
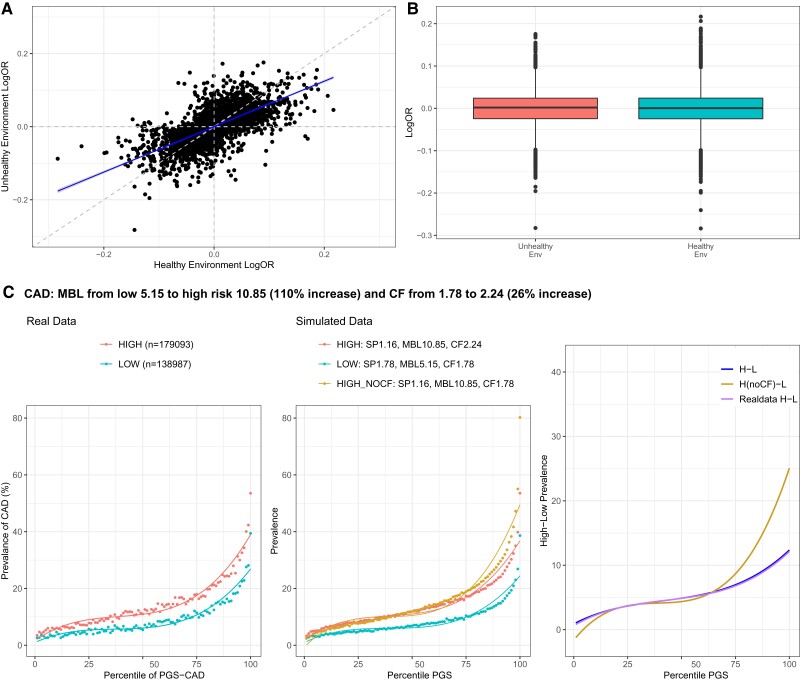
Calibration of the high-risk score. (*A*) Scatter plot contrasting estimated odds ratios for CAD for 2,499 significant SNPs (*P* < 0.001 in discovery data set) in the low (healthy) and high (unhealthy) exposures. (*B*) Boxplots showing the distribution of logOR effect size estimates in two environments. (*C*) The left-hand panel shows the prevalence–risk curve in the high- and low-risk exposures with indicated numbers of individuals. The middle panel shows simulated data with best fits of the MBL, CF, and SP as shown, including a curve (HIGH_NOCF) indicating the effect of not increasing the CF. The right panel shows the deviation between the high- and low-risk curves at each percentile of PGS, clearly indicating for CAD how failure to calibrate overestimates prevalence at high genetic risk. The curve for simulated data (H-L) after calibration closely overlay the curve for real data (Real data H-L) representing the observed difference, whereas the curve without calibration (H(noCF)-L) is over-dispersed. See Supplementary fig. S3, Supplementary Material online for other traits.

For most of the traits considered here, this analysis is complicated by the fact that prevalence is higher in one of the two environments. Since under the threshold liability model, odds ratios are expected to become smaller as the prevalence increases, assuming the same effect sizes in the two environments actually overestimates the cumulative polygenic risk. In fig. 4*A*, we confirm this expectation for CAD effect sizes computed in low (healthy) versus high (unhealthy) exposures: the estimated odds ratios are highly correlated but consistently ∼25–40% smaller for the more sedentary lifestyle in which obesity is more common. Correspondingly, the CF required to produce an optimal fit of the prevalence–PGS curve in the high-risk environment is also 26% larger than that in the low-risk one, given the elevated MBL (fig. 4*C*). Similar results were observed for the majority of binary (disease, obesity, or CA) traits, implying that the observed high-risk curve is consistently below what it would be without calibration.

In fact, CF increase is also required to attain appropriate overall model calibration, without which combining the high- and low-risk curves consistently overestimates the number of cases, as shown in supplementary fig. S3, Supplementary Material online. Our approach produces almost identical calibration to that proposed by Wei et al. (2020) in their study using Platt scaling to provide appropriate calibration for clinical implementation of cancer PGS. Prima facie, these results imply widespread canalization, but they are actually an artifact of the effect of prevalence on PGS estimation under the liability threshold model.

### Evidence for Canalization–Binary Traits

**Fig. 5.**
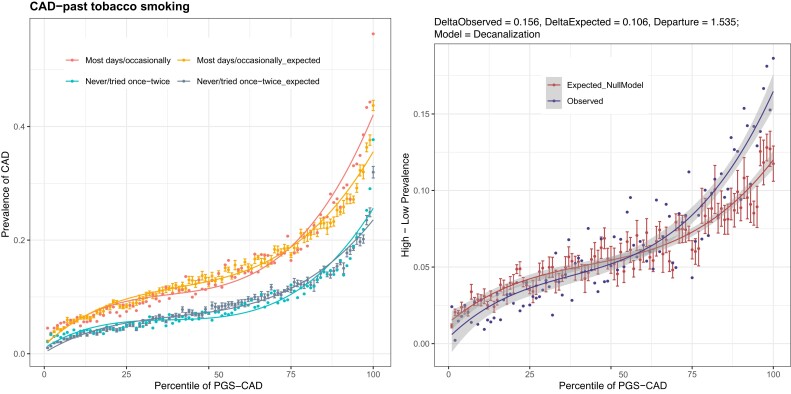
Observed versus expected deviation between environments. (*A*) Prevalence–risk curves CAD for never-smokers and smokers, with expected values modeled from the liability threshold model under the assumption of no PGS×E. Error bars derived from variance estimates of the nonlinear regression. (*B*) The deviation between the expected curves under null and the observed data showing that above the 75th percentile of polygenic risk, the observed data are over-dispersed. Gray bands represent 95% confidence intervals for each curve.

We instead sought evidence for the decanalization in deviations between observed and expected data mostly at the extremes of the prevalence–risk curves. For each environment and PGS percentile, we observe both the prevalence and the mean PGS, and we assume that the liability threshold is constant in each percentile bin and equivalent to that defined by the overall prevalence. This allows computation of least-squares regression that yields the expected prevalence for each bin, as explained in more detail in Materials and Methods. The deviation at the extremes between the expected curves in the two environments, which typically diverge in a monotonically increasing manner, can be compared with the deviations between the two observed curves. Figure 5 shows the example of past tobacco smoking and CAD risk, where never-smokers have the known significant reduction in overall prevalence relative to frequent smokers (blue vs. red curves in fig. 5*A*), but the difference is actually greater than expected under the null of no PGS×E interaction (gray vs. yellow curves), as shown in fig. 5*B*, which plots the deviations as a function of the PGS percentile. When a PGS×E interaction term is included in the regression model, the observed and expected curves overlay almost perfectly, confirming that smoking interacts with genetic risk for CAD to increase prevalence nonadditively. There is, in fact, decanalization of disease in the high-risk exposure. Although we have not directly modeled the dependency of the PGS on prevalence in this framework, we note that the analysis is conservative in so far as the threshold liability model actually predicts that the two expected curves should, if environment-specific weights were used, be closer together than they are in these models, mitigating against seeing decanalization.

To quantify the degree of G×E, for each trait, we generated a rank order of the magnitude of the observed deviations at the extremes (see bar graphs in supplementary fig. S4, Supplementary Material online with a side-by-side estimate of the expected deviations). Deviations at the extremes are quantified by a metric called delta, which is computed as the difference in the right tail deviation versus left tail deviation, where the deviation of the tail is computed as the difference in prevalence between high- and low-risk environments at PGS above or below 2 SD from the mean. Any cases where the difference between these two estimates was more than 2 SDs greater than the mean SD of all of the expected differences is regarded as a candidate instance of decanalization or canalization because the tails are more or less divergent than expected by chance. Suggestive cases were defined where the departure of observed deviations from expected deviations was >±1.3 SDs (top 10%). Given that ∼100 exposures were considered for each trait, only two or three instances are expected to survive this strict criterion, but it should also be noted that for some traits, there appears to be an excess of weak but nonsignificant differences. In supplementary fig. S5, Supplementary Material online, we plot the magnitude of the left-versus-right tail deviation for each trait and exposure, highlighting significant instances of excess.

For CAD, almost all of the deviations are greater at the right tail than the left tail, most being within a few percentage points of the null expectation, but four conditions stand out as instances of decanalization since the worse exposure has an excess of cases at the right tail. These are male gender, smoking status (past and current), father no longer alive, and high average beer consumption. Conversely, having satisfying family relationships, or surprisingly having experienced a long period of DEP, trends toward being protective. For T2D, observed deviations are also almost universally greater than those predicted by the null models but only having family relatives with diabetes approaches significance, possibly implying enhanced risk due to excess familial segregation of genetic risk factors. The IBD analysis confirms significant decanalizing effects for the dietary indicators white versus whole grain bread and adding salt to food while hinting that smoking also exacerbates the genetic risk. All of these instances can be visualized in supplementary fig. S6, Supplementary Material online in the format of fig. 5, as well as through our shiny app, and a full table of significant deviations is provided in table 1 and all other traits in supplementary table S2, Supplementary Material online.

**Table 1.** Canalization versus decanalization model based on the departure of delta observed from delta expected in SD units, for CAD, T2D, obesity, DEP, and IBD.

Trait	Description	Category	DeltaObs	DeltaExpect	Obs-Exp Departure	Model
CAD	Gender	Early life factors	0.276	0.149	3.884	Decanalization
CAD	Father still alive	Familial	0.276	0.192	2.572	Decanalization
CAD	Avg weekly beer and cider	Lifestyle	0.167	0.087	2.450	Decanalization
T2D	Father has diabetes	Familial	0.150	0.081	3.006	Decanalization
T2D	Alcohol intake frequency	Lifestyle	0.149	0.089	2.639	Decanalization
T2D	Nap during the day	Lifestyle	0.199	0.142	2.477	Decanalization
T2D	No. of days vigorous act	Lifestyle	0.102	0.053	2.107	Decanalization
T2D	No. of hours of work in a week	Lifestyle	0.042	−0.006	2.068	Decanalization
T2D	Walk pace	Lifestyle	0.177	0.131	2.002	Decanalization
BMIObesity	Walk pace	Lifestyle	0.290	0.238	2.988	Decanalization
BMIObesity	Duration of walks	Lifestyle	0.061	0.012	2.823	Decanalization
BMIObesity	Mothers age	Familial	0.045	0.002	2.446	Decanalization
BMIObesity	Birth weight	Early life factors	0.082	0.044	2.168	Decanalization
BMIObesity	Maternal smoking at birth	Early life factors	0.100	0.063	2.131	Decanalization
BMIObesity	Mother has diabetes	Familial	0.107	0.070	2.090	Decanalization
BMIObesity	Father still alive	Familial	0.016	0.054	−2.207	Canalization
BMIObesity	Cheese intake	Diet	0.003	0.042	−2.259	Canalization
BMIObesity	Body size at age 10	Early life factors	0.115	0.155	−2.328	Canalization
BMIObesity	Age asthma diagnosed	General health	0.044	0.088	−2.538	Canalization
BMIObesity	Jobs w/heavy physical work	Lifestyle	−0.030	0.033	−3.615	Canalization
WHRObesity	Body size at age 10	Early life factors	0.072	0.005	3.459	Decanalization
WHRObesity	Father has diabetes	Familial	0.064	0.008	2.925	Decanalization
WHRObesity	Loneliness	Psychosocial	0.037	−0.011	2.530	Decanalization
WHRObesity	Seen psychiatrist nerves	Psychosocial	0.031	−0.011	2.203	Decanalization
WHRObesity	Type of accommodation	Socioeconomic	0.042	0.001	2.149	Decanalization
WHRObesity	Number of children fathered	Familial	0.020	−0.020	2.120	Decanalization
WHRObesity	No. of vehicles in household	Socioeconomic	0.028	−0.011	2.064	Decanalization
WHRObesity	Length of mobile phone use	Lifestyle	−0.044	−0.001	−2.267	Canalization
WHRObesity	Time spent driving	Lifestyle	−0.041	0.011	−2.699	Canalization
WHRObesity	Alcohol intake frequency	Lifestyle	−0.061	0.011	−3.767	Canalization
Depression	Duration of vigorous act	Lifestyle	0.034	−0.002	2.426	Decanalization
Depression	Mothers age	Familial	0.037	0.004	2.249	Decanalization
Depression	Tension	Psychosocial	0.006	0.044	−2.532	Canalization
Depression	Avg total household income	Socioeconomic	−0.034	0.003	−2.540	Canalization
IBD	Sibling blood pressure	Familial	0.014	−0.007	2.596	Decanalization
IBD	Bread type	Diet	0.037	0.016	2.554	Decanalization
IBD	Seen psychiatrist nerves	Psychosocial	0.014	−0.006	2.427	Decanalization
IBD	Birth weight of first child	Familial	0.016	–0.0005	2.064	Decanalization

Major DEP is noteworthy for a bimodal distribution of differences between the observed and expected deviations. Various measures of poor exercise habits appear to exacerbate risk, as may not have been breastfed as a baby, but some psychosocial measures such as perceived loneliness, being particularly sensitive to having hurt feelings, and tension may actually be slightly protective despite being risk factors overall. Power for these estimates is undoubtedly reduced due to the relatively low variance explained by PGS_DEP_ and heterogeneity in self-reported DEP. For CA, most of the instances resembling decanalization have to do with the type of work, mode of transportation, and time spent outside and likely indicated responses rather than causality: for example, people with very high EA PGS and who went to college are much more likely to walk to work. On the other hand, this analysis revealed numerous instances of possible canalization, notably for Townsend deprivation index, consistent with observations when considering EA as a continuous trait in the next section (supplementary figs S4–S6 and fig. 7, Supplementary Material online).

### Evidence for Canalization—Continuous Traits

**Fig. 6.**
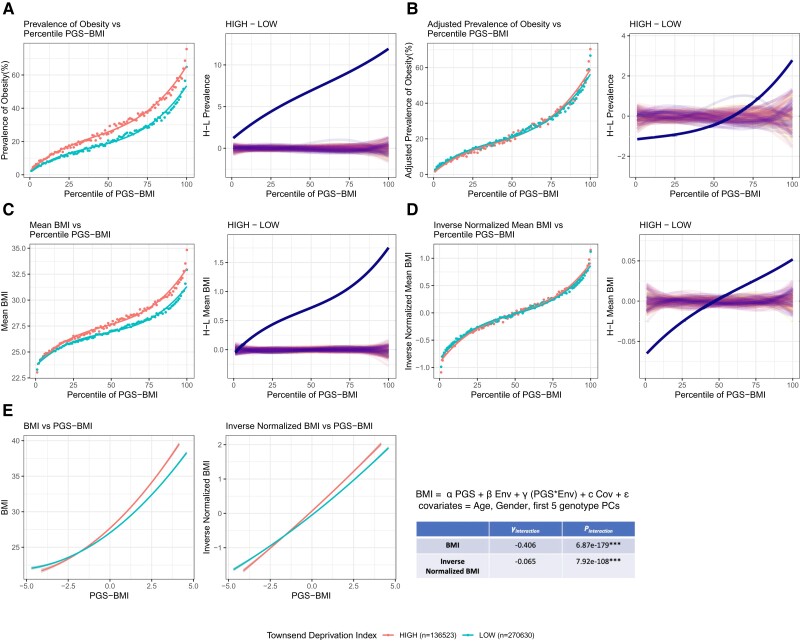
Identification of canalization for a continuous trait. (*A*) Prevalence–risk curves for obesity defined as BMI >30 in the low Townsend deprivation index (TDI) and high TDI. The plot to the right of each panel shows the deviation of the observed curves (solid line) relative to 100 permutations. (*B*) Adjusted prevalence of obesity by redefining the BMI cutoff to give the same overall prevalence in the high TDI as a low TDI environment. The curves are closer but still deviate monotonically. (*C*) The mean BMI versus percentile PGS curves; plotting the mean BMI in each percentile bin. (*D*) Inverse-normalized mean BMI centered around zero in both environments, also showing that high TDI has increased variance relative to low TDI. (*E*) Linear regression of individual trait and PGS scores for raw BMI and inverse-normalized BMI in the two environments, showing highly significant crossing of line means, along with estimates of the interaction term from the linear regression model.

The continuous traits BMI, WHR, and EA present opportunities to document G×E without the complication of the dependence of PGS on differences in prevalence under the liability threshold model. We took four approaches. (1) Modeling each trait categorically as above but adjusting the threshold of obesity or higher education to ensure the same prevalence in the high and low exposures. (2) Regressing the observed trait value for each person on their PGS and comparing the slopes between the two exposures—this analysis is noisy but yields conclusions comparable with those of the next two approaches. (3) Computing the trait mean in each percentile bin of the PGS and evaluating the deviations at the left and right tails, in this case taking the difference between the exposures as the measure of G×E without the need to generate expected curves. (4) Performing inverse normalization of the cumulative distribution function of the percentile means, which is more conservative and focuses on deviations at the extremes. In each case, we adjusted the trait mean for age, gender, and the first five genotypic principal components. Figure 6 shows the example of the influence of Townsend deprivation on BMI, which overwhelmingly provides evidence for decanalization, namely genetic risk combines with low socioeconomic status (high Townsend deprivation, red) to increase the likelihood of weight gain synergistically.

**Fig. 7.**
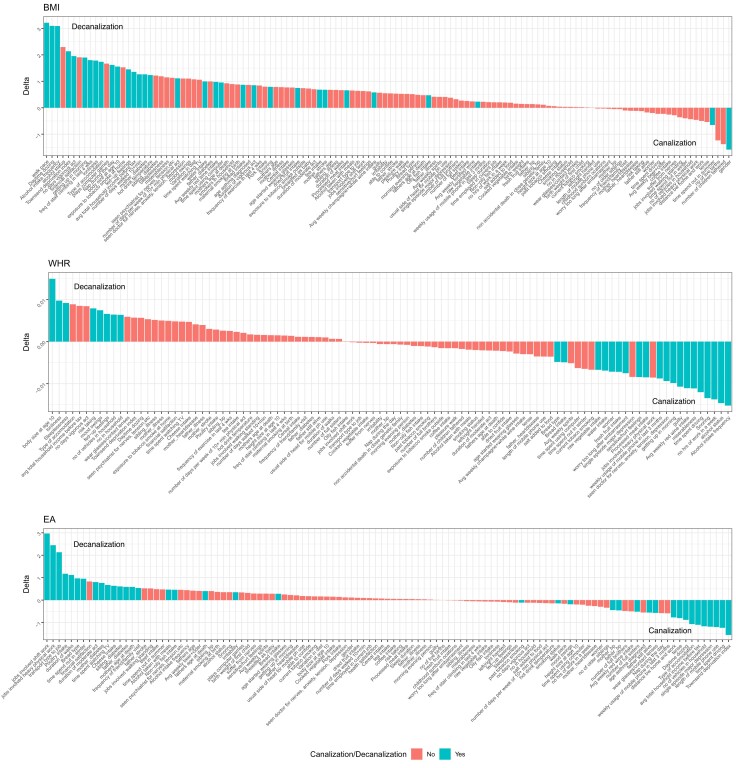
The rank order of (de)canalization for the continuous traits. Each plot shows the deviation between the left and right tails (delta) for the given exposure for BMI, WHR, and EA on unscaled measures of the traits. Blue bars indicate that the deviation was judged to be beyond 2 SDs of the mean on the inverse-normalized data scale, implying decanalization to the left (positive delta) or canalization to the right.

The model fits for the trait mean–PGS curves as a function of the SP, MBL, and CF were computed using nonlinear least-square regression (see Materials and Methods). Typically, the CF is larger in the trait-increasing environment, which might imply canalization as noted above, but offsetting this observation was a general decrease in the SP (supplementary fig. S7, Supplementary Material online), which implies an increased deviation between the left tail and midpoint. Although there is no reason to expect the nonmodifiable component of risk to reduce in the increasing environment, the model fitting indicates that all three parameters covary. Consequently, we used the inverse-normalized trait values to contrast the two curves, ensuring that the MBL is set to be the same in the two environments. This both smooths skew in the curves (fig. 6*E*) and ensures that differences in the shapes of the curves are due to the SP and CF and independent of the MBL. It yields two highly correlated estimates of (de)canalization: the delta between the upper and lower tails, which we require to be greater than that observed in 100 permutations of the environmental labels, and the variance of the inverse-normalized trait values, which are plotted against one another in supplementary fig. S8, Supplementary Material online (see Materials and Methods for details). These criteria were used to define instances of greater or lesser than expected deviation between the high and low curves (decanalization and canalization, respectively) for all available exposures, which are then highlighted in the rank order for the three traits in fig. 7, along with right versus left tail deviations in supplementary fig. S9, Supplementary Material online.

For BMI, the evidence for decanalization is pervasive: 82 of the 126 contrasts have positive deviations, 32 of which are significantly larger than expected after inverse normal transformation. The top three exposures are slow walking pace, taking daytime snoozes, and (low) alcohol consumption. Multiple different measures of activity, mood, and socioeconomic status all seem to combine with genetic risk to increase BMI more than additively. Only two possible instances of canalization characterized by a reduction of the deviation at the right tail are self-reported, which are spending a lot of time outside, and gender: although men tend to have higher BMI, the elevation is only seen in the bottom three-quarters of the PGS_BMI_ range.

For WHR, the deltas are an order of magnitude lower than for BMI, implying a modest impact of most exposures on this measure of body shape, but intriguingly there is more evidence for canalization than decanalization: 20 versus eight exposures. The most convincing instance of the latter is body size at age 10, in direct contrast with the data shown in fig. 1*D* for categorical BMI-defined obesity, implying that genetic impacts on WHR tend to manifest in children and exacerbate with age. In addition, in contrast to BMI, high alcohol consumption associates with elevated WHR, but the influence dissipates as genetic risk increases. Similarly, longer working hours and more time spent driving have a greater tendency to elevate WHR for individuals with low polygenic propensity to gain central adipose.

As with the categorical assessment, EA shows extensive G×E of both types. Instances where EA is elevated across the genetic spectrum but become progressively larger as PGS_EA_ increases once again are likely to indicate responses to higher education. They include the type of job and transportation but also certain dietary indicators (whole grain vs. white bread, cheese consumption). Some exposures simply reflect differences in variance of the PGS without a strong overall effect. For example, EA is less associated with genetics in people who consume more chicken or use their mobile phones more. A low Townsend deprivation index and experiencing a single episode of DEP are both more likely to elevate EA for those with a low genetic propensity, whereas the top quartiles of the PGS_EA_ distribution have little difference in outcome relative to exposure. Consequently, since the exposure with the higher prevalence overall has a less genetic variance, by our definition these are cases of canalization (although as with CA, since the high-risk exposure is actually high deprivation, this is perhaps just as well regarded as a case of decanalization relative to the more healthy environment). It will be interesting to evaluate how these effects decompose into cognitive and noncognitive aspects of genetic propensity (Demange et al. 2021). Left and right tail deviations for the continuous traits are shown in supplementary fig. S9, Supplementary Material online.

### Genetic and Environmental Correlations

The PGS distributions are rarely identical between exposures. Rather, in general, there is a slight elevation of the mean PGS in the “bad” (high-risk) exposure group, as expected since the disease incidence is higher. The SDs of the PGS do not show any trend and, for the most part, have low deviations. These values are reported in supplementary table S3, Supplementary Material online. To evaluate whether the elevated PGS explains higher prevalence, we computed the effect of substituting a PGS with the observed mean and variance into the best-fit SP, MBL, and CF models for the low exposure. The most extreme deviations are notably due to the exposure of family members to the disease, suggesting that the elevated PGS in those exposures reflects shared genetic rather than environmental risk. However, even in the cases with the highest observed increase in mean PGS, approximately 10%, the expected increase in prevalence was never more than 10% of the observed increase. Consequently, most effects related to environmental exposures are somewhat independent of PGS distribution.

Nevertheless, there is a strong correlation between increased genetic risk and an increased proportion of individuals in the high exposure group (Young et al. 2019). This is seen in supplementary fig. S10, Supplementary Material online for T2D as a function of Time spent watching TV, Townsend Deprivation Index, and walking pace. The upper plots show strong linear correlations of the ratio of high-to-low-risk individuals in each percentile of polygenic risk. These results are explained readily by the right-shift of the high relative to low-risk curves. Although striking, again, since the increase in PGS only explains a minor proportion of the increase in risk, the genotype–environment correlation only contributes to a minor fraction of deviation between the risk profiles.

A priori, there is no reason why a PGS should differ across environments when it is computed without adjustment for those environments, but the systematic observation that it does suggests a couple of possibilities (Dudbridge and Fletcher 2014). One is that the increase in genetic effects in the high-risk condition is asymmetric with the result that more individuals with high-risk disposition cross a threshold of liability, and hence the average risk in the high-risk environment is slightly higher than in the low-risk one. This could, for example, occur if myriad small individual G×E interactions tend to operate more in the direction of increased risk with exposure: variants that increase the risk of obesity with a more sedentary lifestyle may have correlated directional increases in sedentary individuals. Another possibility is reverse causation, where the onset of a disease or condition (which is more likely in higher PGS individuals) leads to a higher likelihood of individuals adopting the high-risk lifestyle (for example, becoming more sedentary).

## Discussion

Under persistent stabilizing selection, which is thought to be the predominant mode of selection acting on gene expression and many traits (Hodgins-Davis et al. 2015; Sanjak et al. 2018), some theories suggest that genetic architectures may evolve to ensure that most individuals are close to the optimum (Hermisson et al. 2003; Geiler-Samerotte et al. 2018). This process is known as canalization, which is the evolution of genetic interactions that suppress the generation of extreme phenotypes. Our study provides the first evidence that this process may be widespread in relation to the genetic predisposition to human disease. Assuming the parsimonious genetic interpretation of the PGS×E interactions, namely that elevated baseline risk gives rise to larger combined odds ratios as the PGS increases, the divergence in prevalence between the tails is sometimes less than expected given the overall difference in prevalence between two exposures. On the other hand, particularly in relation to BMI-related obesity, CAD, and T2D, the prevalence of the condition in individuals with high-risk exposures is more often higher than it is expected to be, implying decanalization (Gibson 2009; Gibson and Lacek 2020). A particularly interesting observation is that BMI and WHR have generally opposing evidence in relation to canalization, not just overall, but for specific exposures such as gender and alcohol consumption. This may relate to the contrasting strength of stabilizing selection on cognitive and metabolic traits throughout mammalian evolution and, more recently, in the human lineage.

Decanalization occurs when a novel environment releases genetic variance, leading to an elevated number of individuals with extreme phenotypes in the low and high polygenic risk categories. On the face of it, the increasingly elevated prevalence in those with both genetic and environmental exposure implies that substitution effects are increased under these conditions. In this sense, there is decanalization on the purely additive scale. As a matter of epistemology, odds ratios are a useful statistical interpretation that describe the patterns in the data but are blind to the molecular mechanism. Genes operating inside cells certainly see environmental effects, but their activity is blind to the population mean, and there is no molecular genetic reason to expect that absolute allelic substitution effects would vary as a function of the baseline risk. Stated another way, it may have been common for the baseline that is acted on by the genetics to be constant across environments, and for the environment to increase risk additively, irrespective of PGS. This was sometimes observed, but the overwhelming characteristic of the cases described here is that the environment increases the baseline exposure that is then multiplied by the PGS to yield the observed prevalence–risk relationships. Our categorical analyses are also complicated by the dependence of odds ratios on the prevalence of the trait (Wray and Goddard 2010), which increases as the genetically MBL of risk increases, deflating allelic effects in the perturbing environment. We describe a variety of approaches to adjust for this complication, by deriving the expected prevalence of disease and comparing deviations at the tails, and by modeling on the continuous scale, but acknowledge the need for further theoretical development of PGS–prevalence modeling.

The existence of PGS×E contrasts with accumulating evidence regarding obesity, for example, where individual SNP-by-environment interactions that are too small to survive strict GWAS multiple comparisons, even though they contribute to broad-sense heritability (Abadi et al. 2017; Tyrrell et al. 2017; Nagpal et al. 2018; Sulc et al. 2020). A bias toward such effects operating in the same direction can easily generate deviations in the genetic architecture that may lead to modification of the prevalence–PGS relationships (Marigorta and Gibson 2014). On the other hand, as noted, intuition suggests that in some cases, reverse causality is more likely to explain the observations, namely that those who develop a condition are more likely to transition to a different environment. Diabetes, for example, may impact mobility and diet, although in general, it is not obvious why such effects would not just cause a linear shift of the curve since the MBL should not be affected. Two possibilities are (1) individuals with the highest genetic susceptibility experience more severe disease, which in turn elevates the likelihood of behavioral change, and (2) pleiotropic genetic contributions impact the polygenic risk for both the trait and the likelihood of experiencing the exposure. Alternatively, transgenerational sharing of the environmental exposure may exist, whereby environmental factors such as household income, bread choice, and type of job, although measured for participants in middle age, are actually proxies for related factors culturally transmitted from parents that act earlier in life. Differences in heritability measured within families relative to that expected from GWAS also led Mostafavi et al. (2020) to conclude that indirect genetic effects lead to more pervasive G×E than is generally acknowledged.

Our results have implications for the modeling of the evolution of the genetic architecture of complex traits. The GWAS-enabled recognition of the pervasive omnigenetic contributions to phenotypic variance led Simons et al. (2018) to propose that differences in the number and effect size distributions for different traits are mediated by the mutational target size and fitness impact under a model of stabilizing selection with extensive pleiotropy. Evidence that genetic effects are modified in fluctuating environments and specifically buffered in suboptimal conditions, suggests more scope for modeling this effect on the degree of polygenicity. Equally importantly, quantification of the degree of canalization and pleiotropy may support renewed modeling of the evolution of canalization (Hallgrimsson et al. 2019; Takahashi 2019) and its compatibility with observed effect size distributions across environments.

Three possible mechanisms are most likely to contribute to canalization. One is the existence of epistatic interactions (G×G) that suppress the additive component of the additive variance and have been postulated to evolve under intense stabilizing selection with sufficient mutational variance (Wagner et al. 1997). The second is the emergence of G×E interactions in the perturbing environment that reduce individual allelic effects, a mechanism that could evolve to reduce the impact of environmental noise effectively. The third is the existence of cryptic genetic variation, namely allelic effects at some loci that only manifest in one or other of the environments, with the result that the PGS captures different amounts of the genetic variance, indirectly influencing the variance explained by the PGS. More complex developmental and physiological systems may be prone to evolve canalization under the constraints of networks that must be resistant to mutations and environmental insults (Siegal and Bergman 2002), but molecular evidence for this remains elusive.

A limitation of this study is the lack of independent validation. Some results may be replicable by large consortium studies for specific traits, although it is not clear how findings would be impacted by meta-analysis of contributing cohorts or by specific cultural attributes in different settings. It should also be noted that using large cohorts entails risks related to hidden biases, such as healthy volunteer bias in the UKB (Fry et al. 2017), self-report bias, especially for behavioral traits (Xue et al. 2021), as well as sex-differential participation bias (Pirastu et al. 2021). Given the low portability of PGS across ancestry groups (Chen et al. 2015; Martin et al. 2017), particular attention to the development of ancestry-aware scores will be needed before the application of our approach to other large public health genomic studies, including different ancestry groups (Vujkovic et al. 2020). Internally, we validated most findings by performing all analyses with two different sets of SNPs ascertained at stringent and permissive cutoffs. For BMI, we also adopted the PRS–CS Bayesian approach (Ge et al. 2019) to PGS development since it increases the variance explained, and just as with the inclusion of more SNPs in the prune + threshold settings reported here, the PGS×E were not only affirmed but became more robust.

Irrespective of causality, our findings have practical implications regarding the implementation of polygenic risk assessment for personalized medicine. Although logistic regression approaches are beginning to offer joint genetic–environmental assessments (Rudolph et al. 2018, Isgut et al. 2021), they are unlikely to capture nonlinear interactions, and new methods may need to be developed. More generally, the pervasiveness of apparent PGS×E interactions highlights how important it is to account for the environment in offering individual risk assessments (He et al. 2019). Thus, someone in the 25th percentile for PGS_T2D_ with a pretax household income <£52,000 has the same probability of developing diabetes, around 4%, as someone in the 75th percentile with a higher income. Similarly, for the influence of maternal diabetes. These data confirm widespread mismatches between our genome and the contemporary environment. Health disparities are shaped in part by genetic differences across populations, but to the extent that the environmental factors described here differ among localities, interaction effects reinforce the complex interplay of nature and nurture in shaping the distribution of disease on a fine geographic scale (Haworth et al. 2019; Lakhani et al. 2019).

## Materials and Methods

### The UKB Cohort

The UKB is a large population-based cohort consisting of ∼500,000 individuals, recruited between 2006 and 2010 at 22 assessment centers spread across the UK (Sudlow et al. 2015). The participants, aged 40–69 at recruitment, completed baseline questionnaires about lifestyle, physical measures, medical history, and general health, as well as providing biological samples (blood, urine, and saliva) for genetic, proteomic, metabonomic analyses, and biomarker identification (Elliott et al. 2008). In addition, the UKB has also generated data fields to indicate the first occurrence of a set of diagnostic codes for a wide range of health outcomes across self-report, primary care, hospital inpatient data, and death data, all mapped to a three-digit code of International Classification of Disease (ICD-10). In this study, we downloaded the genotype and phenotype from the UKB under application number 17984. The imputed genotype data (named v3) released in May 2017 for ∼ 96 million markers was downloaded (Bycroft et al. 2018). After selecting bi-allelic variants with imputation score > 0.9, minor allele frequency >1%, Hardy–Weinberg equilibrium *P* > 10^−10^ and <5% missing rate, a total of 8,063,507 SNPs were available for analysis.

The phenotypic data for 10 complex traits were downloaded and classified as categorical: obesity defined by BMI and WHR, T2D, CAD, CA, DEP, and IBD, CD, and UC and continuous: BMI, WHR, and EA in years. These included both self-reported questionnaire data and ICD-10 codes. The complete list of UKB data fields used, exclusion criteria, and the number of cases and controls obtained are provided in supplementary table S5, Supplementary Material online. In addition, only self-reported White British individuals (*n* = 408,925) were included in this analysis.

### Environmental Exposures

For the environmental exposures, we downloaded 151 data fields from the UKB broadly categorized into diet, lifestyle, socioeconomic, early life factors, familial factors, psychosocial factors, and general health. The phenotypic values of 55 fields were numerical such as “pieces/bowls/cup per day” for fresh fruit, bread, cereal, raw vegetable, tea, coffee, water intake; “number of glasses” for average weekly red wine intake, average weekly beer intake, average weekly champagne intake; “hours per day” for time spent outdoors in summer, time spent driving, time spent watching TV, sleep duration, duration of walks, number of days/week of vigorous physical activity, Townsend deprivation index score. An additional 98 fields had ordinal values in the form of responses such as: “Never,” “less than once a week,” “once a week,” “2–4 times a week,” “5–6 times a week,” “once or more daily,” “special occasions only,” for beef, processed meat, lamb, cheese intake, alcohol intake frequency; “yes,” “no,” “occasionally” for current tobacco smoking; “slow,” “average,” “brisk” for walk pace, and so on. Each exposure was dichotomized to represent high and low exposure, either by the ceiling of the mean phenotypic value for numerical fields or based on answers for ordinal fields. Furthermore, individuals having missing phenotypic values, or who answered “Do not know” or “Prefer not to answer” were excluded. Supplementary table S4, Supplementary Material online contains the list of data fields used for 151 environmental exposures, the criteria of defining high and low-risk environmental groups, and their sample size. Five exposures with very high PGS×E were excluded from computations since they are almost certainly consequential to disease onset: major dietary changes in the last 5 years, long-standing illness, overall health rating, health satisfaction, and shortness of breath.

### GWAS and Calculation of PGS

Supplementary table S5, Supplementary Material online provides the references and sample sizes of the European-based GWAS summary statistics for nine complex traits used for this analysis. First, we performed SNP pruning on the summary statistics using “–indep-pairwise” function in PLINK (Purcell et al. 2007) to filter out variants within LD r2 > 0.2 within a 1000 kb window. The numbers of variants obtained after pruning are also listed in supplementary table S5, Supplementary Material online. Next, we selected variants at two significance thresholds: (1) genome-wide significance level of *P* < 5×10^−8^ and (2) at *P* < 0.001. These were used to compute PGS with the “–score” function in PLINK at the two significance thresholds. Supplementary fig. S11, Supplementary Material online shows the distribution of the effect sizes and the PGSs for the traits. Further, for BMI, we also evaluated our results using the PRS–CS Bayesian approach (Ge et al. 2019) of estimating posterior SNP effect sizes under continuous shrinkage (CS) priors using GWAS summary statistics and a reference LD panel. The estimated effect sizes from PRS–CS were then used to construct PGS using PLINK.

### Assessment of PGS×Environment Interactions

To detect deviations in the prevalence–risk relationship across environments, we compared the shapes of the prevalence–risk curves, given that the underlying PGS have essentially a similar distribution in both environments. For each of the 151 environmental exposures, dichotomized into low and high groups, the prevalence of disease versus percentile PGS plots were generated. Next, the difference between the prevalence of the high and low curves (H-L) along the polygenic risk gradient was computed. A monotonically increasing trend of the (H-L) curve would indicate the case of decanalization, implying that as the genetic risk increases, the impact on disease increases and more so in the bad exposure environment. Conversely, a constant (H-L) curve along the risk gradient would imply that the curves are simply shifted, and there is no observed decanalization. For consistency, high in all cases was defined simply as the environment with the elevated risk overall and is not taken to imply that one or the other exposure is intrinsically high risk. This occasionally leads to counterintuitive results where the inference of canalization or decanalization is flipped (note that decanalization with respect to one exposure is canalization with respect to the other). A notable example is Townsend Deprivation Index with College Attainment, where the H exposure is low TDI since it has a very slightly elevated prevalence of CA, and hence canalization is reported in fig. 7 and supplementary figs. 4, 5, 6, 9, and 10, Supplementary Material online, yet intuitively since high TDI is the poor socioeconomic exposure, this is discussed as a case of decanalization in supplementary fig. S2, Supplementary Material online and the text.

To evaluate this phenomenon quantitatively, permutation analysis was performed by randomly selecting individuals to high or low exposure groups, keeping the sample size of the high and low groups the same as the real data in the UKB (as given in supplementary table S4, Supplementary Material online). The prevalence versus percentile plots were generated for the randomly selected individuals, and H-L curves were computed. In general, the H-L curve from random permutations should follow a nonmonotonic trend around zero. Therefore, to establish the evidence of interactions, the following conditions were required. (1) The derivative of the H-L curve be consistently either greater or lesser than 0, and the median of the derivative distribution is always higher than that generated from 100 random iterations. This ensures a significant monotonicity of the curve. (2) The absolute value of the linear slope of H-L curve (equivalent to the delta between the upper and lower tails) must be greater than that generated from 100 randomly permuted data. Once the evidence of interactions was established, its degree was defined as delta, the right versus left-tail deviation in prevalence at two SD units from the PGS mean (supplementary fig. S12, Supplementary Material online shows the departure of delta observed from delta expected for 151 exposures and all binary traits summarized, where a positive departure represents decanalization and a negative departure represent canalization. The dashed lines are at +/− 2 SD units and +/− 1.3 SD units indicating conservative and suggestive thresholds respectively). More details of this and subsequent methods are provided on our Shiny app.

### Modeling Canalization—Continuous Traits

A simple measure of canalization for continuous traits is a difference in the slope of the regression of each individual’s trait value on their PRS, in the two environments, adjusted for the covariates age, gender, and population structure (the first five principal components of the genotypes):

(1) Trait=α.PGS+β.Env+γ.(PGS×Env)+c.Cov+ε

where the significance of the α, β, and γ terms represent main effects of the PGS, environmental category, and their interaction (the canalization term), respectively, while ε is the residual error, assumed to be normally distributed with a mean of zero. Inverse normal transformation of the trait typically removes any curvature (see fig. 6*E*) and is a more conservative analysis. Despite a very high significance of γ in many cases (supplementary table S6, Supplementary Material online), implying a difference in slope between the environments, the scatter of points is very large, so we prefer to evaluate canalization from the distribution of trait (or inverse-normalized trait) means in each percentile of the PGS.

Then, to infer (de)canalization we compare the deviations between the high and low mean trait-PGS curves. First, nonlinear least-square regression is implemented with the *nls* function in R, fitting each individual’s trait as a function of a SP, MBL, PGS, and CF:

(2) Trait=SP+MBL×exp(PGS/CF)

This model captures the intuition that a PGS represents the cumulative log odds ratio of the effect of SNPs on the trait. The PGS is centered to a median of zero, with an observed SD, implying that the expected trait value at the 50th percentile where PGS = 0 is the MBL plus SP. For example, the average BMI in a population maybe 27, of which 15 units are independent of genetics (the SP), and 12 units are modifiable by genetics (the MBL). In this case, if the SD of the PGS is 1, then CF = 4 adjusts the exponential effect to fit the observed typical trait value to those observed; for example, one SD unit above or below the mean would be 30.41 (15 + 12*e^1/4^) and 24.35 (15 + 12*e^−1/4^) respectively. However, if the MBL in the obesogenic environment is 14 (two points higher) without changing the SP, then the expected values would be 32.98 and 25.90. If they are actually 32.48 and 26.21, then the CF would need to be 4.5, implying that the polygenic effects are actually slightly reduced in this adverse environment, given the overall increase in BMI. Alternatively, if most of the environmental effect was independent of the genetics, a slight increase in MBL to 12.25 and elevation of the SP to 16.75, without changing the CF, would also result in an expected BMI of 32.48, one SD unit above the mean, but in this case, the value one SD unit below the mean would be slightly higher at 26.29. The *nls* regression generally estimates different values for all three parameters (SP, MBL, and CF) in each environment, indicating that all three parameters must be adjusted to obtain optimal curve fits. Note that since the PGS itself does not generally have a variance of 1, the proportion of individuals at any particular risk level is also a function of the observed variance of the PGS. The mean square *R*^2^ estimate of the models approximates the SNP heritability, namely the variance explained by the PGS, which is also typically higher in the elevated risk environment.

Even though the CF is generally larger in the high environment, the SP is generally lower, consistent with a greater slope of the simple linear regression model. Since these two results, increase in the linear term but effective decrease in PGS as the CF increases, are contradictory with respect to the overall impact of genetics, we instead perform the high versus low comparison on the inverse-normalized trait scale to assess evidence for canalization. Constraining the MBL to be the same in both environments (set to the observed value in the low environment) ensures that prevalence is the same at the median risk, and hence that only the SP and CF determine the shape of the mean invNormTrait-PGS curve. Given the measured parameters from the data, as well as the observed variance of the PGS, σ^2^, the median trait value is estimated as [SP + MBL], the mean trait value E{Y} as [SP + MBL×exp(σ^2^/2CF^2^)], and the variance of the trait value Var{*Y*} as [SP^2^ + MBL^2^×exp(2σ^2^/CF^2^) + 2×SP×MBL×exp(σ^2^/2CF^2^)− (SP + MBL×exp(σ^2^/2CF^2^))^2^]. At both the right and left tails, 2 SD units greater or lesser than the PGS mean, we compute the expected mean trait value as E{Trait|PGS > 2σ} = [SP× (1−CDF(2,0,1)) + MBL× (1−CDF(2−σ/CF),0,1)× exp(σ^2^/2CF^2^)]/(1−CDF(2,0,1)), replacing 1−CDF with CDF for E{Trait|PGS <−2σ}. These values are computed in the high and low environments, yielding expected right and left tail deviations, the difference between which is delta (fig. 7 and supplementary fig. S9, Supplementary Material online). The magnitude of delta is then compared with that computed from 100 permutations of the environment labels of the individuals. We infer decanalization if delta is greater than all permutations and canalization if it is lesser than all permutations and observe that this measure is highly correlated with the Var{Y}, which yields a concordant measure of (de)canalization (supplementary fig. S8, Supplementary Material online).

### Modeling Canalization—Binary Traits

Estimation of the expected deviation between environments is complicated for categorical (case–control) traits because under the threshold liability model, odds ratios are a function of prevalence. Consequently, in an environment with higher prevalence, genetic effect sizes are expected to be smaller, and the PGS estimated from the full data set will tend to overestimate the variance in the high environment and underestimate it in the low environment. The observed required increase in CF can be interpreted as simply correcting for this bias. We thus again turn to comparison at the extremes to define instances of canalization but compare the observed deviations with those expected from modeling the prevalence–PGS relationships. We also expect that deviations at the extreme will be usually be associated with PGS×E influences on risk throughout the distribution.

We assume that within each percentile of risk, individuals have a liability, μ_i_, that is a function of their PGS and environment (μ*_i_* = *a* * PGS + *b* * Env + *c*), and a prevalence, *P_i_* = 1 − CDF(*N*(*μ_i_*,1) < ***t***), where the threshold ***t*** is constant and estimated from the overall liability distribution, ***t*** = CDF^−1^(1 − *P*_overall_) for *N*(0,1). That is to say, the overall liability distribution is a collection of 100 liability distributions for which *μ_i_* increases as the PGS increases in an environment-specific manner. Since we observe the mean PGS and prevalence in each percentile bin, and μ*_i_* = *a* * PGS + *b* * Env + *c* = ***t*** − CDF^−1^(1 − *P_i_*), linear regression is used to estimate *a*, *b*, and *c* from the 200 data points of each percentile bin in environments 0 and 1. These values are then used to estimate the expected prevalence in each bin, E{*P_i_*} = 1 − CDF(*N*(μ*_i_*,1), ***t***) and compared with the real data in the left and right tails, as well as with a model that includes a PGS×Env interaction term.

Departure from the null was computed as delta-observed (the difference between the right and left tail deviations in risk in the two environments) minus delta-expected (the mean difference between the right and left tails computed by 10 iterations of sampling from *N*(*μ_i_*,SD), where SD is the residual error of the linear model), divided by the mean SD of delta-expected across all exposures for the trait. That is to say, we computed the difference between the observed and expected deviations between the tails and scaled it by the magnitude of the SD of the expected effect. Statistical significance of potential decanalization was then defined as cases where the observed departure from the null was more than two SD units larger than the expected deviation. This is slightly more conservative than using the exposure-specific deviations, which may be under-estimated in some cases. Suggestive cases were defined with the departure of delta observed from delta expected under null greater or lesser than ±1.3 SD units (top 10%). Table 1 shows the departure (in SD units) of exposures which passed the conservative threshold for CAD, T2D, obesity, DEP and IBD and supplementary table S2, Supplementary Material online shows the departure (in SD units) for all 151 exposures and binary traits. Supplementary fig. S4, Supplementary Material online shows the delta observed versus delta expected under null along with departure in SD; supplementary fig. S5, Supplementary Material online shows the left versus right tail deviations for all exposures and binary traits.

Since some exposures are relatively uncommon, the error in the observed prevalence can be large, so we also required that the overall high-low prevalence curve be outside the 95% confidence interval of the computed expected deviation under the null (fig. 5). In fact, this criterion suggests considerably more cases of PGS×E than the strict definition defined by deviation at the tails. Furthermore, in each case of presumptive canalization (less deviation than expected) or decanalization (more deviation), fitting the linear regression to estimate *a*, *b*, *c* with an interaction term for PGS×E completely abrogated the deviation between observed and expected, confirming the significance of the interaction effects.

## Supplementary Material

msac053_Supplementary_Data

## Data Availability

The results are explored readily and can be downloaded from the R shiny: https://canalization-gibsonlab.shinyapps.io/rshiny/. Code is at GitHub under account https://github.com/sn-GT/Canalization.
