# Supplementary material for: Canalization of the Polygenic Risk for Common Diseases and Traits in the UK Biobank Cohort
Source: Mol Biol Evol. 2022 Mar 11;39(4):msac053. doi: 10.1093/molbev/msac053 (PMC9004416; doi:10.1093/molbev/msac053)

Supplementary Figure S1

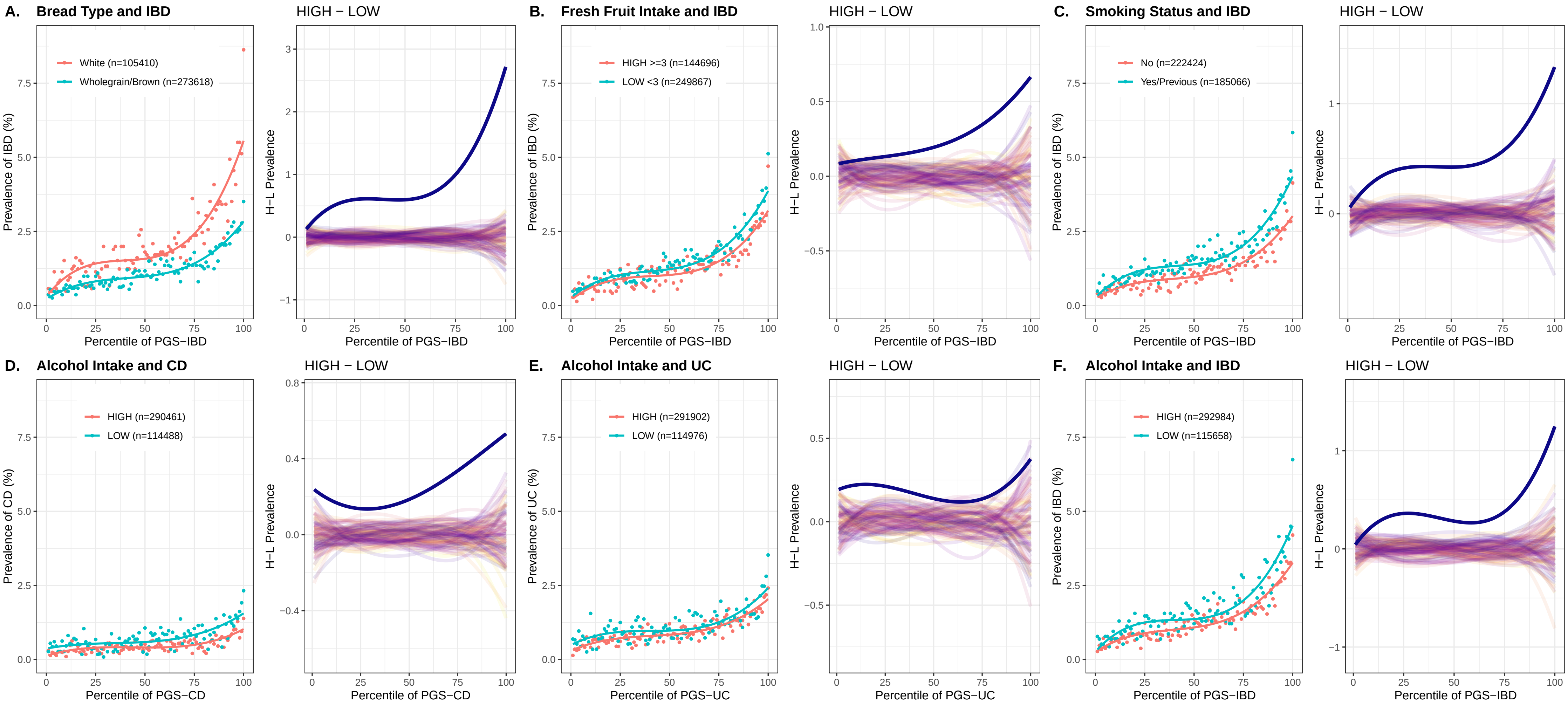

Supplementary Figure S2

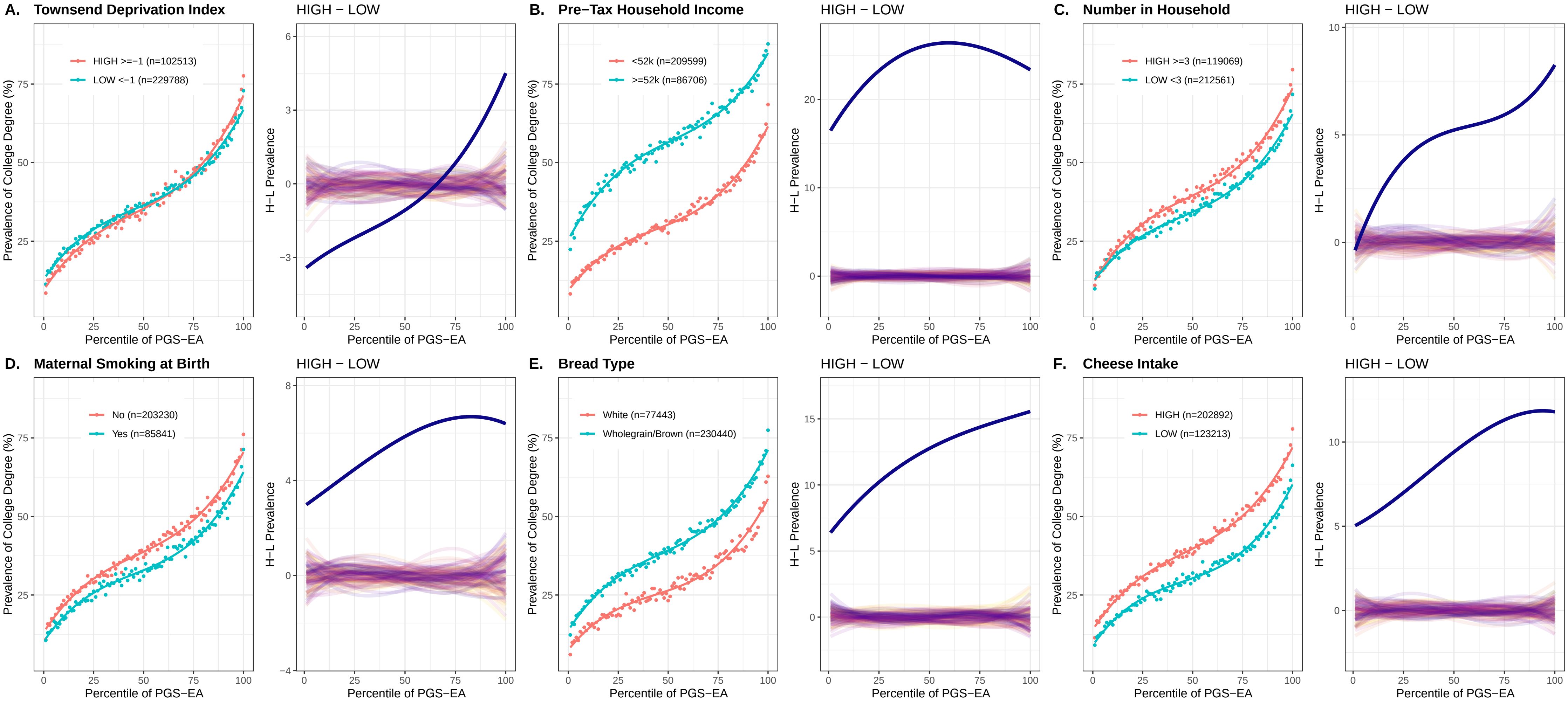

Supplementary Figure S3

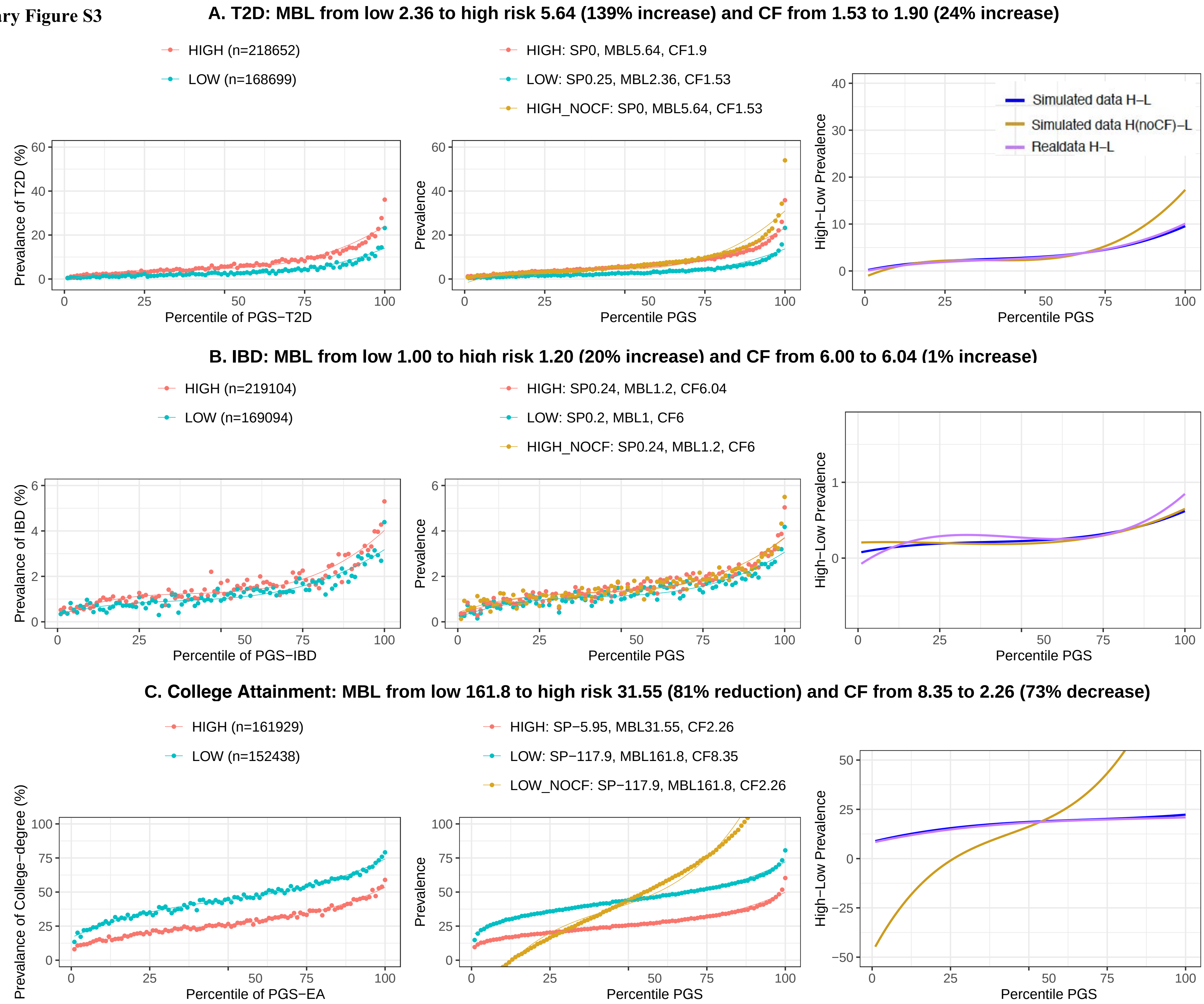

Supplementary Figure S4

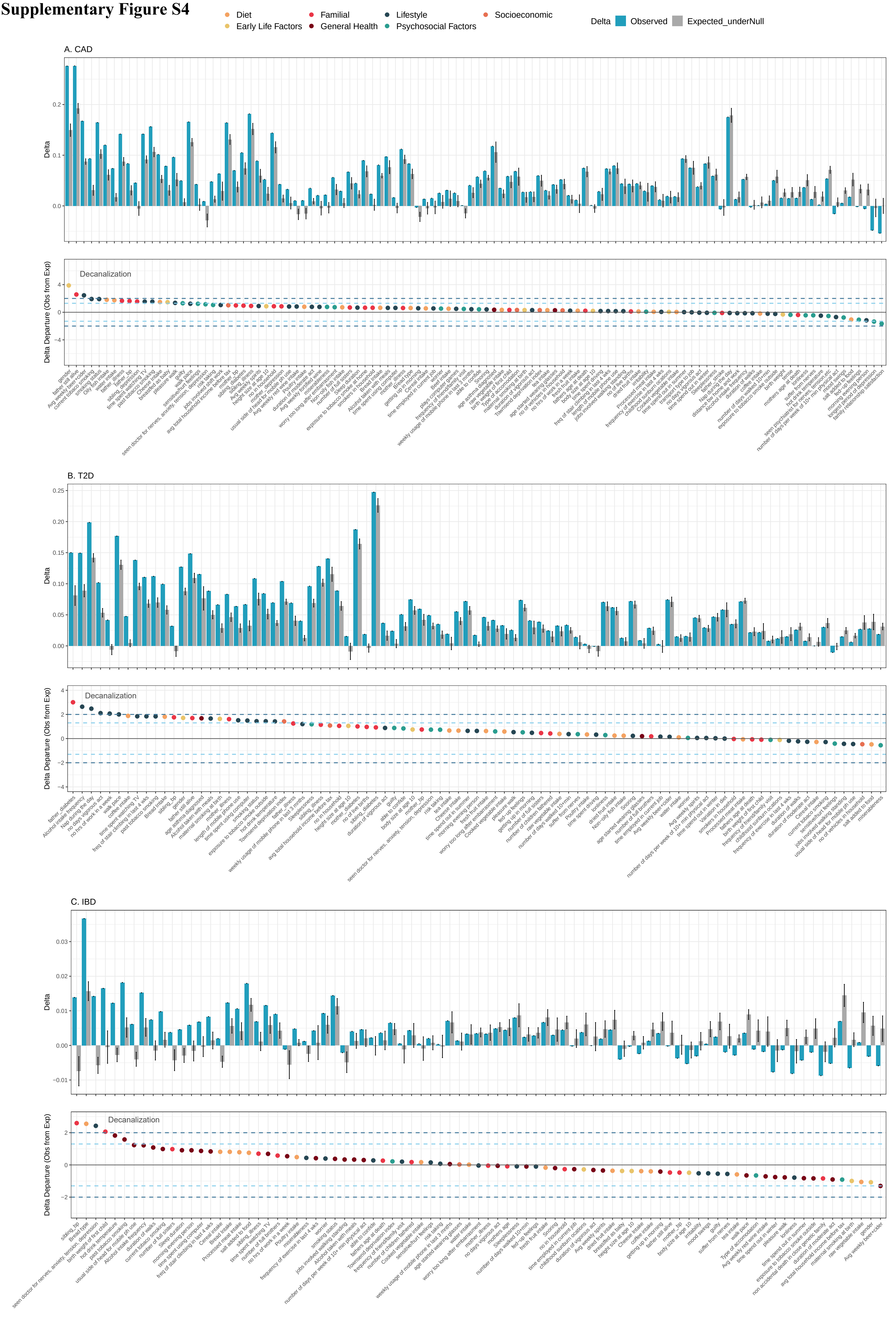

Supplementary Figure S4

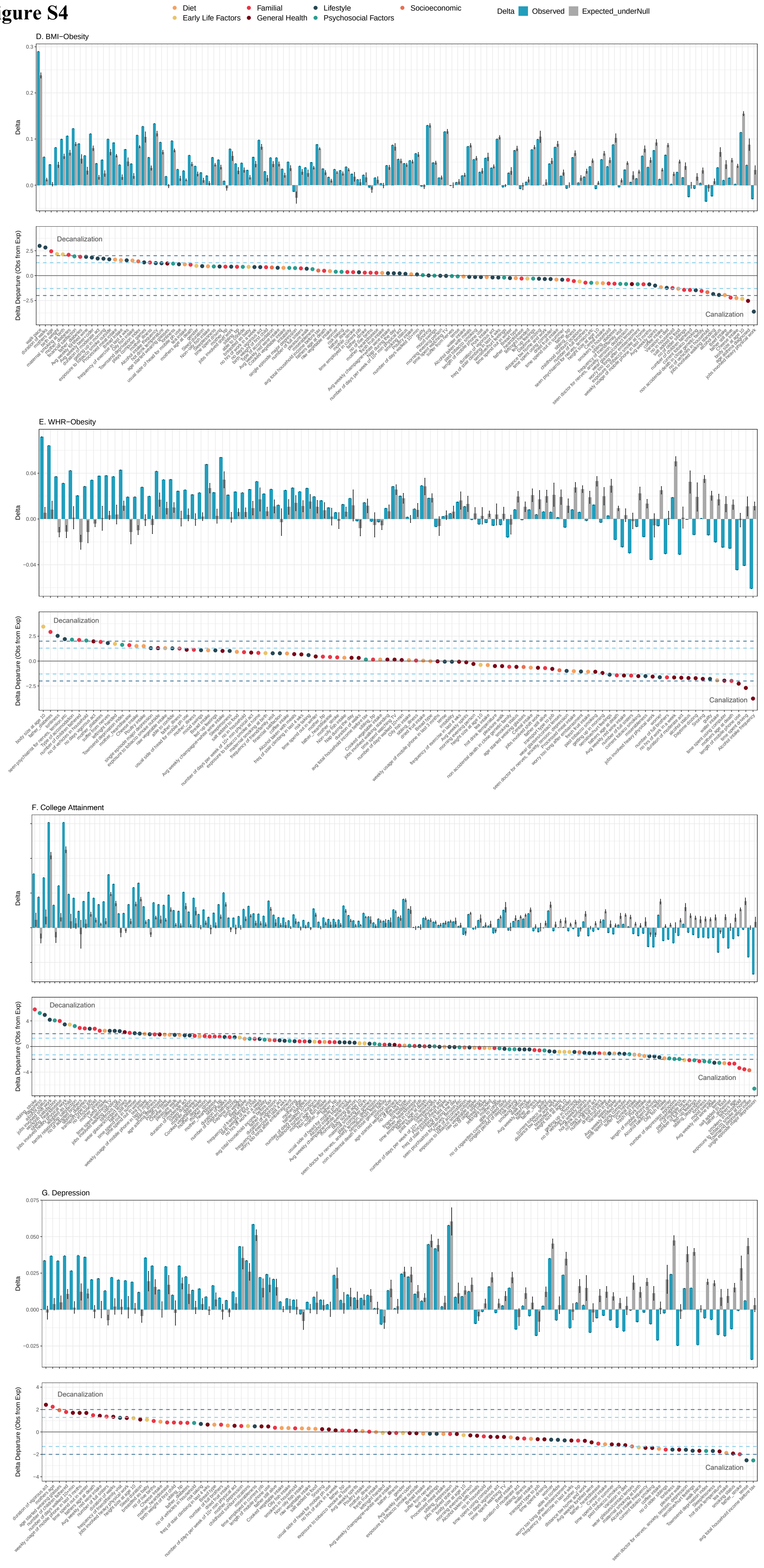

Supplementary Figure S5

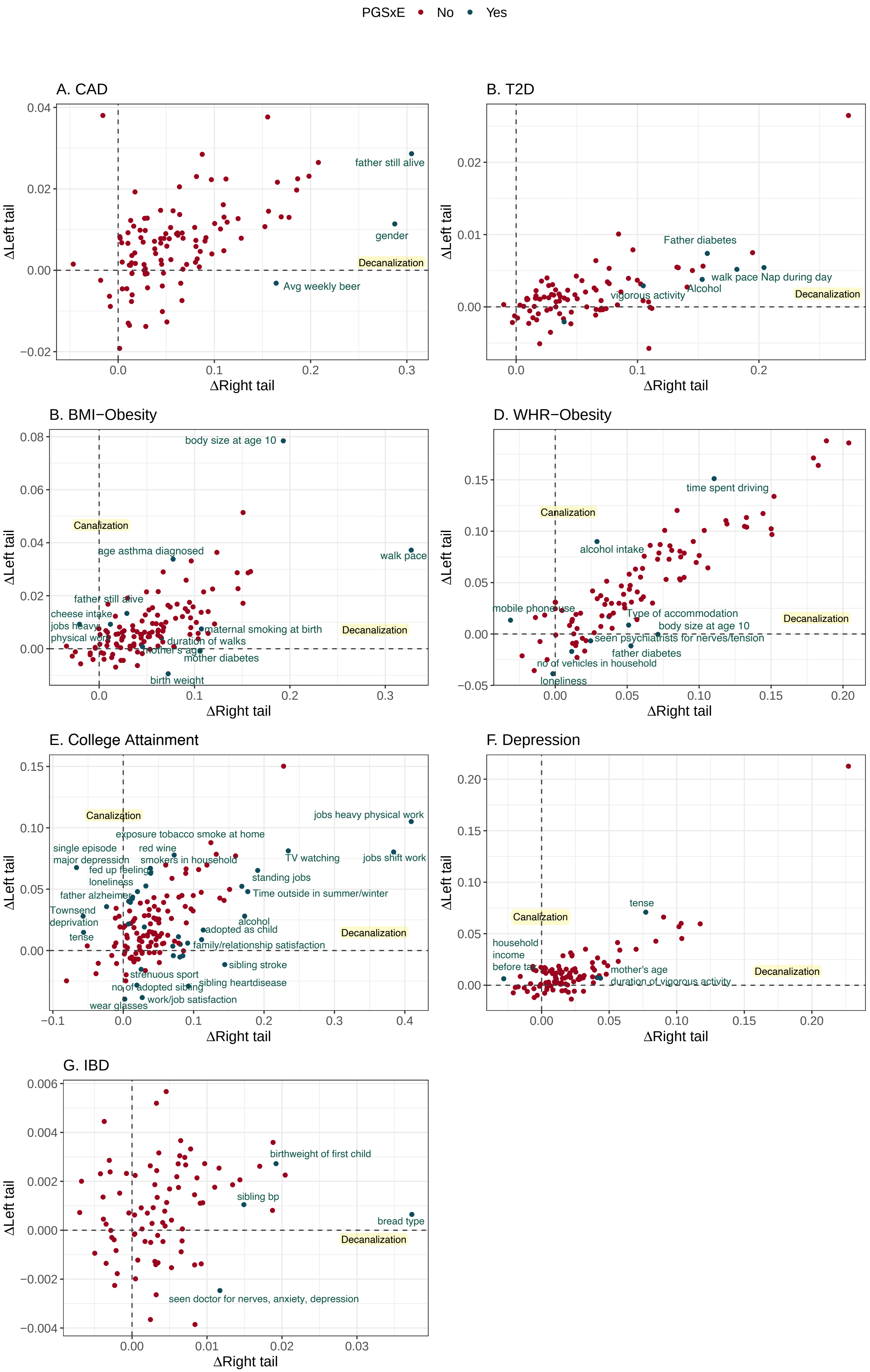

Supplementary Figure S6

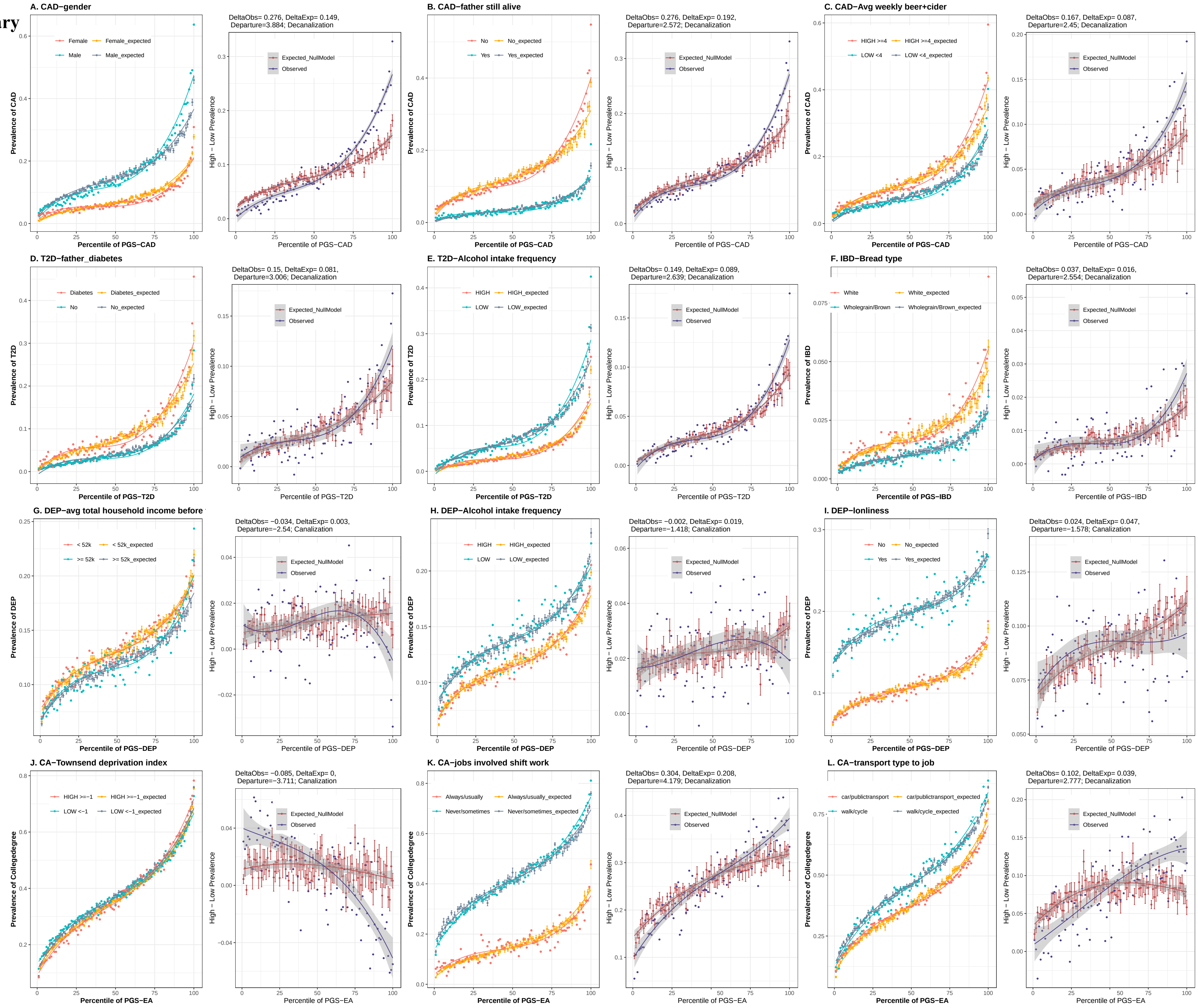

Supplementary Figure S7

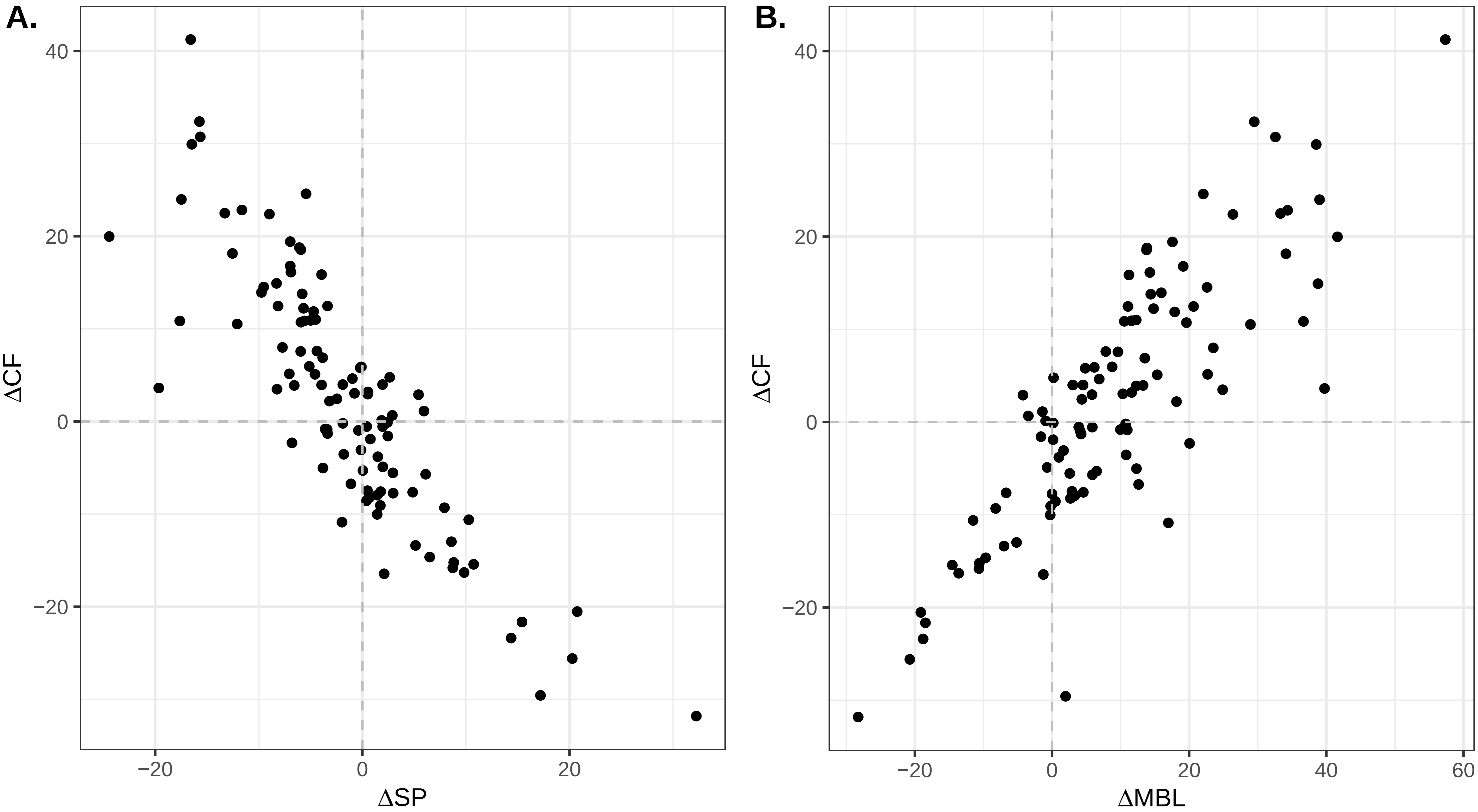

Supplementary Figure S8

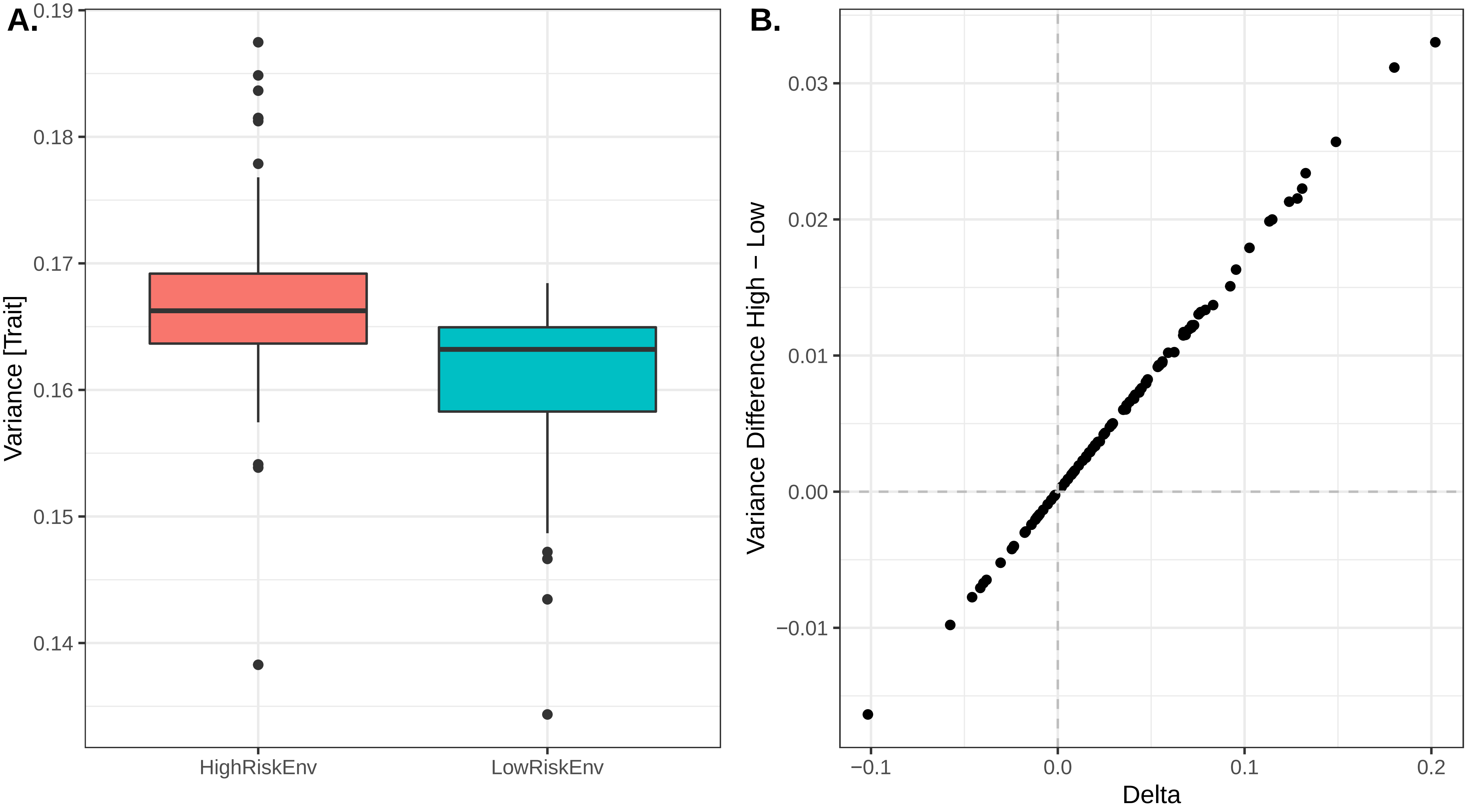

Supplementary Figure S9

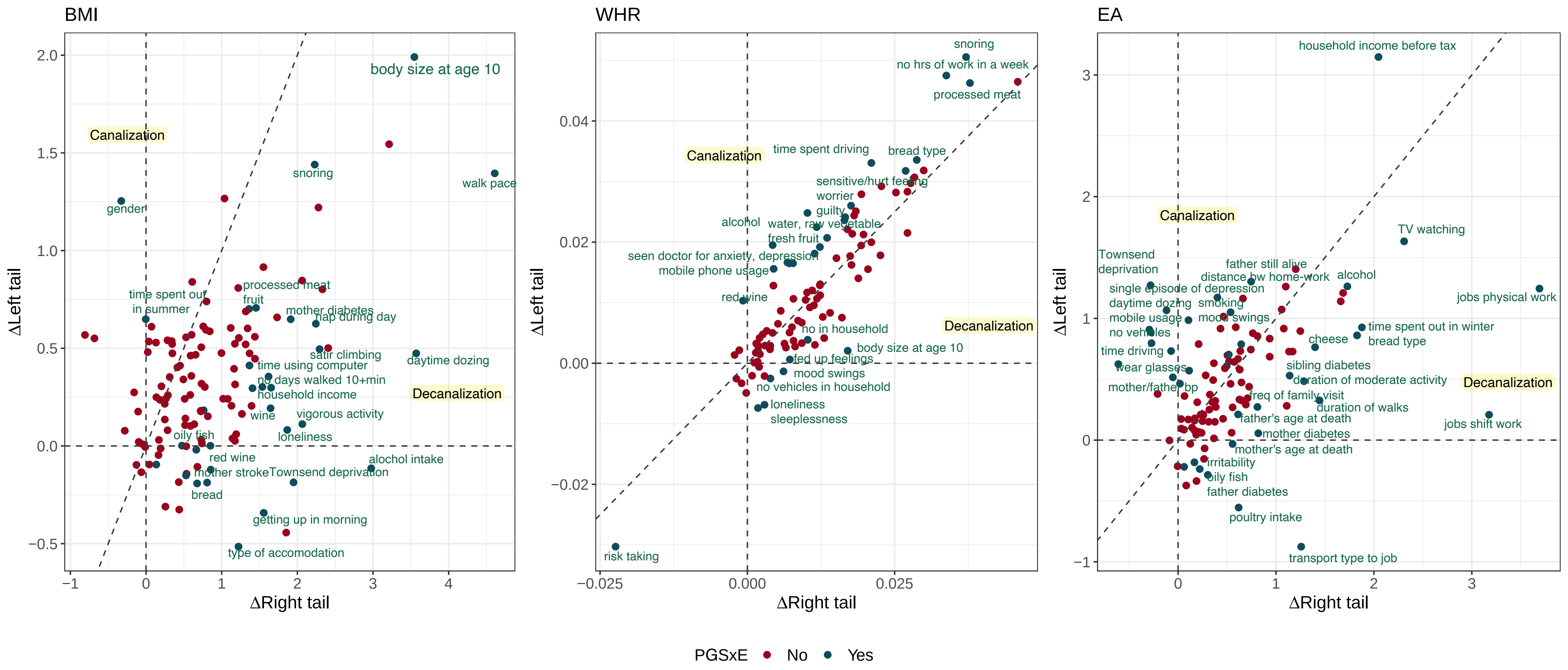

Supplementary Figure S10

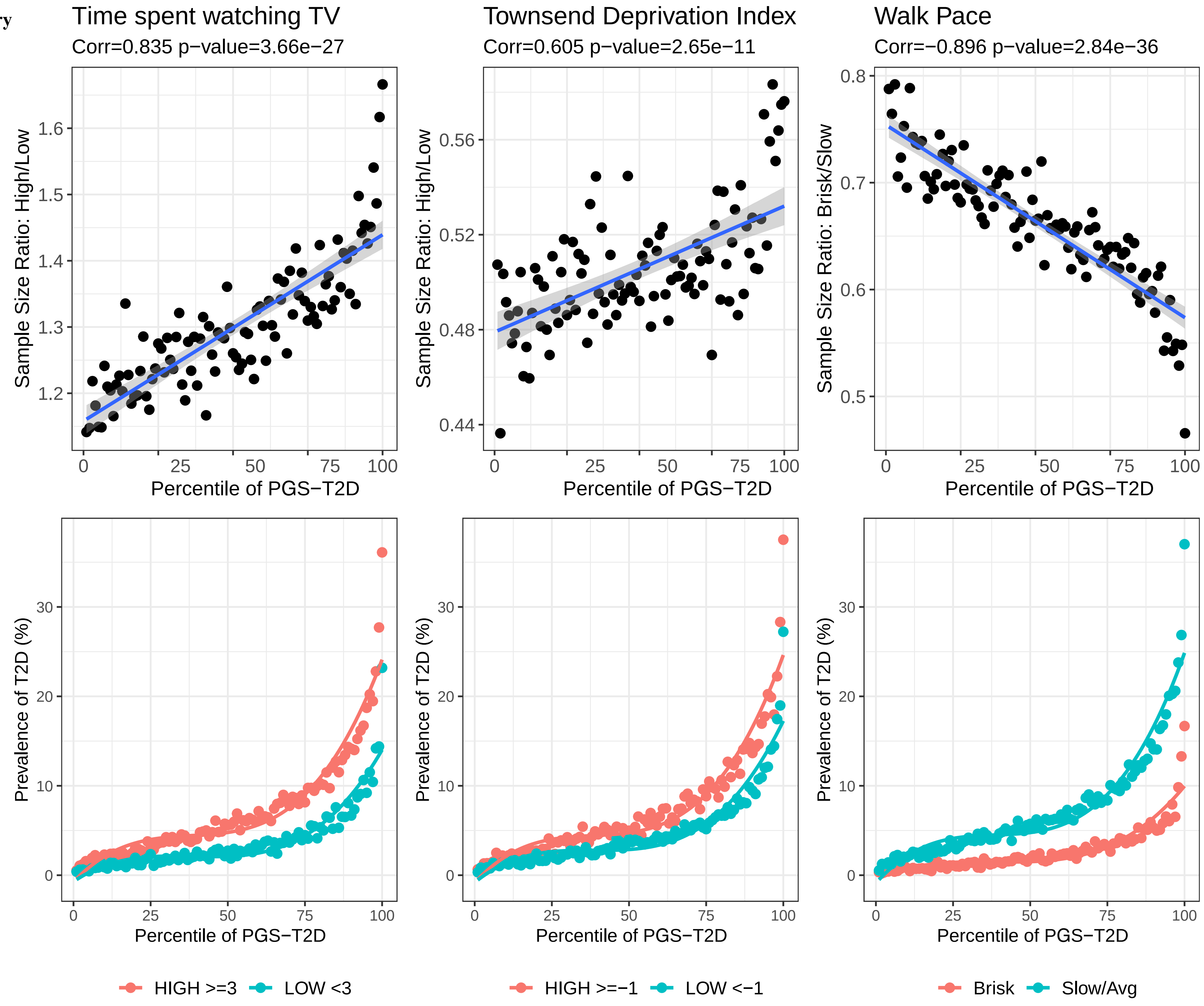

Supplementary Figure S11

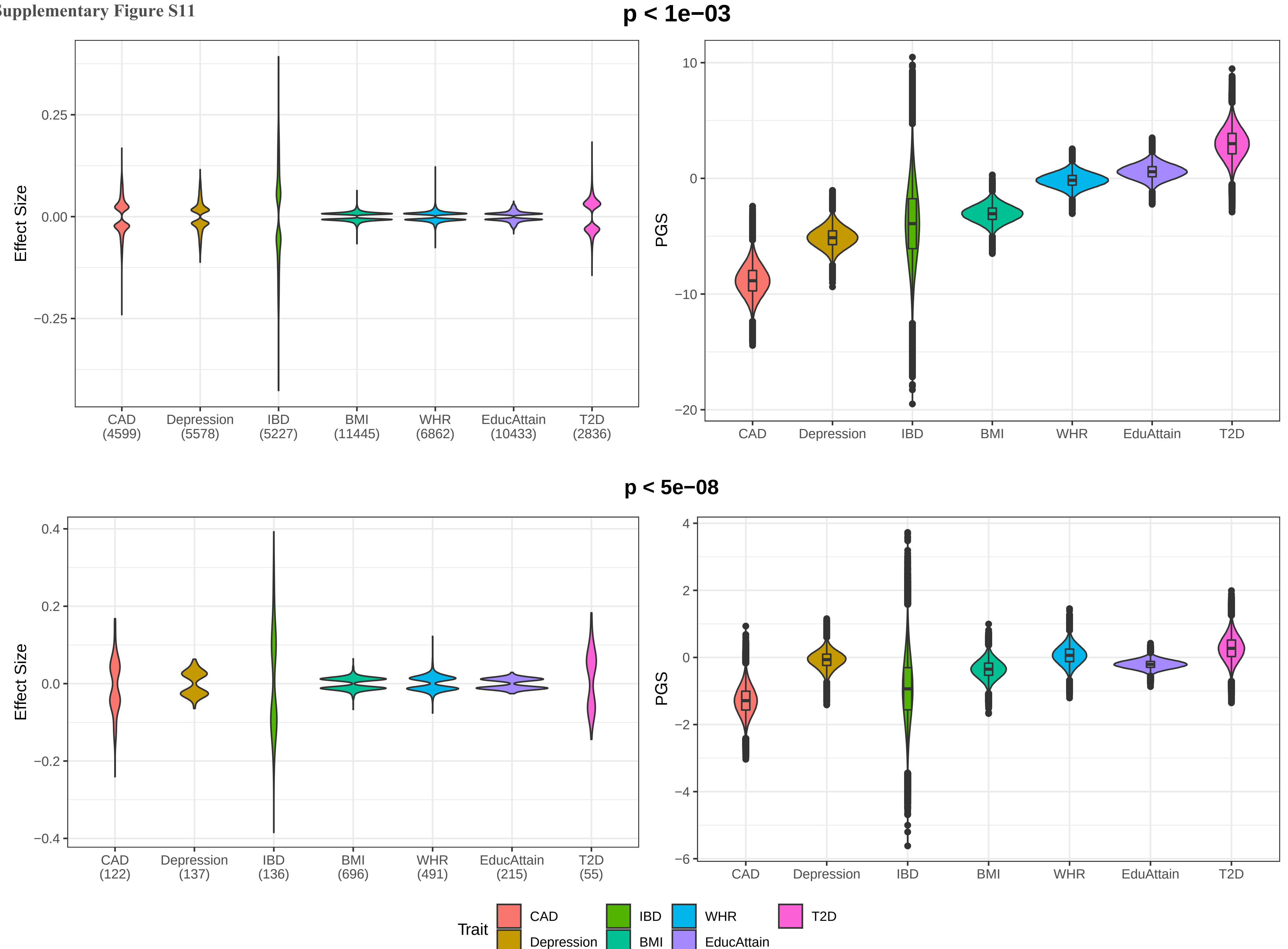

Supplementary Figure S12

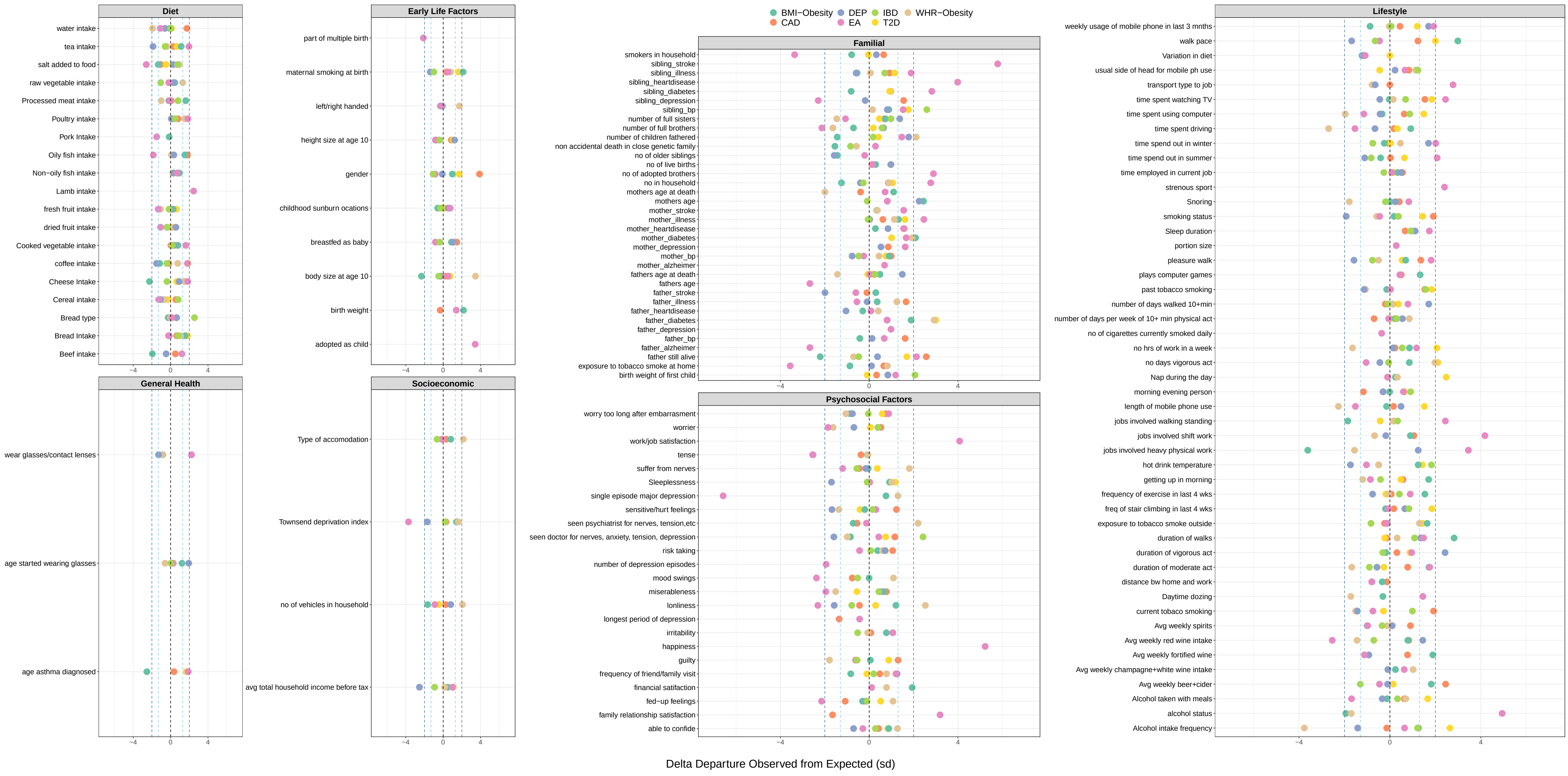

Supplement: msac053_Supplementary_Data [file msac053_supplementary_data.zip › SupplementaryFigures_Nagpal.pdf]
